# Reflection of Fast Neutrons from Water^1^

**DOI:** 10.6028/jres.063A.006

**Published:** 1959-10-01

**Authors:** Martin J. Berger, John W. Cooper

## Abstract

The backscattering of fast (0.3, 1, 3, 6, 9, and 14 Mev) neutrons from a semi-infinite water medium has been calculated by the Monte Carlo method. The information obtained includes the joint angular and spectral distribution of the reflected neutrons, the dependence of the number albedo and energy albedo on the source energy and obliquity, and the contributions to the albedo of successive orders of scattering. The spectra were calculated down to epithermal energies (~0.5 ev). The results for each case are based on the analysis of 3,000 neutron histories, generated with the use of an IBM–704 computer. In the random sampling, elastic scattering from hydrogen and oxygen, inelastic scattering from oxygen, and absorption due to *n-α.* and *n-p* processes were taken into account. The cross sections for some of these interactions are not well known. Parallel calculations with different assumptions about the cross sections were made in order to estimate how sensitively the albedo depends on the cross sections. The paper includes a self-contained description of the Monte Carlo method, its application to the calculation of radiation diffusion and in particular to the neutron albedo problem. Emphasis is placed on the technique of correlated sampling which makes possible an accurate estimate of albedo differences resulting from different assumptions about the cross sections. The random sampling computations were supplemented by analytical calculations of the single-scattering albedo. This was useful for an understanding of the Monte Carlo results because a considerable fraction of the reflected neutrons return after only one collision.

## 1. Introduction

### 1.1. Statement of the Problem

Neutrons that are incident on an extended medium make one or more collisions with nuclei in each of which they are deflected and lose a fraction of their energy. Thus they perform a “random walk” until they are either absorbed or re-emerge from the medium with reduced energy. The probability that an individual neutron will eventually re-emerge from a semi-infinite medium (i.e., be reflected) is called the albedo, or the differential albedo if the energy and/or direction of the reflected neutron are specified.

The purpose of this paper is threefold: (1) To calculate the differential albedo and related quantities for neutrons incident on a semi-infinite water medium; (2) to give a self-contained exposition of the methods of calculation used (Monte Carlo, single-scattering analysis); (3) to analyze how the albedo depends on various factors, particularly the cross sections for elastic and inelastic scattering and absorption.

### 1.2. Background

Exact calculations of the neutron albedo have so far been limited to situations in which the slowing down of neutrons could be disregarded. With this limitation, and with further restrictive assumptions, Halpern, Lueneburg, and Clark [1]^2^ have given an analytic solution based on transport theory. A more general solution of the one-velocity problem is also available from Chandrasekhar’s [2] theory of radiative transfer. Fermi [3] and more recently Grosjean [4] have given approximate solutions which are in good agreement with the more rigorous calculations.

When the complicated energy-dependence and directional dependence of the collision processes must be taken into account, an exact analytical treatment becomes all but impossible. Spinney [5] has given an approximate treatment based on age theory which is applicable mainly to energies lower than those considered in the present work.^3^ Another possible approach would be to incorporate the exact one-velocity treatment of reference [2] into an energy-group scheme similar to the multigroup diffusion schemes used in reactor calculations. This has not been attempted so far.

The Monte Carlo method recommends itself as the simplest and most promising approach. It has the advantage that the complexity of the calculations is not essentially increased by adding to the number of variables or by introducing complicated cross sections. Increased detail merely creates a data-handling problem which requires a computer with sufficient capacity. A disadvantage of the Monte Carlo method is its purely numerical character which makes necessary a very careful scrutiny of the results before one can understand their significance. We have found that such an analysis is greatly facilitated by parallel analytical calculations of single scattering. In spite of its promise, the Monte Carlo method has not yet been used extensively for albedo calculations. Some results for the backscattering of 0.89-Mev neutrons from water have been given by Foderaro and Obenshain [6], but their work was mainly concerned with transmission through slabs.

One reason for the scarcity of Monte Carlo applications to the albedo problem appears to be the lack of adequate information on neutron collision cross sections, which may have discouraged extensive numerical work.^4^ For few if any elements (other than hydrogen) are the cross sections known in the detail and with the accuracy desirable. Only the knowledge of the total collision cross section is in definitive form for most elements. Information about the contributions of different processes to the total cross section is reasonably adequate. Much less is known about differential cross sections. The angular distribution of elastic scattering deflections—which is highly anisotropic at high energies—is not well known for many elements. Even less is known about inelastic scattering. There is uncertainty as to the nuclear levels which are excited, the relative excitation probabilities, and the angular distribution of the inelastic scattering deflections.

We have chosen water as the medium in our calculations because the cross sections for hydrogen and oxygen are known better than those of most other elements. In addition to being of interest in their own right, the results for water will also be typical of those expected with other hydrogeneous substances. Thus the present work should provide guidance for future calculations for media such as concrete which are held up now by lack of cross section data.

## 2. Variables of the Problem

**Figure 1:**
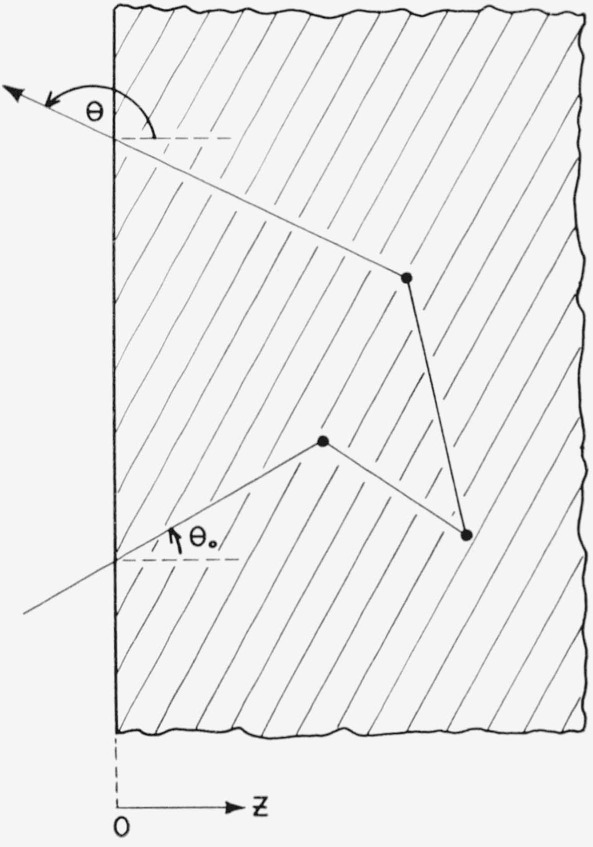
Neutron trajectory.

We consider the reflection of neutrons from a semi-infinite medium occupying the region *z*≥0. Figure 1 shows a typical trajectory of a neutron which is incident on the medium with obliquity *θ*_0_ and which is reflected with obliquity *θ.* These obliquities are angles between the direction of motion of the neutron and the positive *z*-axis. Thus *θ*_0_ lies between 0 and *π*/2, and *θ* lies between *π*/2 and *π.*

We denote by *A*(*E,θ;E*_0_,*θ*_0_) sin*θdθdE* the probability that a neutron incident with energy *E*_0_ and obliquity *θ*_0_ will be reflected from the semi-infinite medium, after one or more scatterings, with an energy between *E* and *E+dE* and an obliquity between *θ* and *θ+dθ.* The function *A*(*E,θ;E*_0_,*θ*_0_) is called the *differential albedo.* Alternatively, instead of regarding the albedo as a probability, we may consider it to be the ratio of the average number of neutrons reflected to the number of neutrons incident. Thus if a beam of *N*_0_ neutrons is incident with energy *E*_0_ and obliquity *θ*_0_, and if *N*(*E,θ*) *sinθdθdE* neutrons are reflected on the average, then

$$A(E,\theta ;E_{0},\theta_{0})=\frac{N(E,\theta )}{N_{0}(E_{0},\theta_{0})}.$$ (2.1)

By integrating the differential albedo one obtains the *number albedo A_N_* (ratio of the number of all reflected neutrons to the number of incident neutrons):

$$A_{N}(E_{0},\theta_{0})=\int_{0}^{E_{0}}dE\int_{\pi /2}^{\pi }\sin\theta d\theta A(E,\theta ;E_{0},\theta_{0}).$$ (2.2)

Similarly, the *energy albedo A_E_* (ratio of the average reflected to the incident energy) is defined as

$$A_{E}(E_{0},\theta_{0})=\int_{0}^{E_{0}}dE\int_{\pi /2}^{\pi }\sin\theta d\theta \frac{E}{E_{0}}A(E,\theta ;E_{0},\theta_{0}).$$ (2.3)

We shall have occasion to consider the probabilities of reflection after exactly 1, 2, … *n* collisions. The corresponding albedos will be indicated as *A*^(^*^n^*^)^(*E,θ;E*_0_,*θ*_0_), *A_N_*^(^*^n^*^)^(*E*_0_,*θ*_0_) and *A_E_*^(^*^n^*^)^(*E*_0_,*θ*_0_), *n*=1, 2, …. The case *n*=1 (single-scattering albedo) is particulary important.

## 3. Neutron Cross Sections of Hydrogen and Oxygen

### 3.1. Integral Cross Sections

The most important parameter characterizing the neutron transport process is the mean free path between collisions, λ(*E*). It is inversely proportional to the number N of target nuclei per unit volume and the total collision cross section *σ*(*E*),

$$\lambda (E)=\frac{1}{N\sigma (E)}•$$ (3.1)

The reciprocal of the mean-free path (the attenuation coefficient) is additive for a homogeneous mixture of different nuclei. For water,

$$\frac{1}{\lambda_{H_{2}O}}=\frac{1}{\lambda_{H}}+\frac{1}{\lambda_{\mathrm{Ox}}}=N_{H}\sigma_{H}(E)+N_{\mathrm{Ox}}\sigma_{\mathrm{Ox}}(E),$$ (3.2)

where $N_{H}=6.69\times 10^{22}{\mathrm{cm}}^{-3}$ and $N_{\mathrm{Ox}}=3.34\times 10^{22}{\mathrm{cm}}^{-3}$.

**Figure 2:**
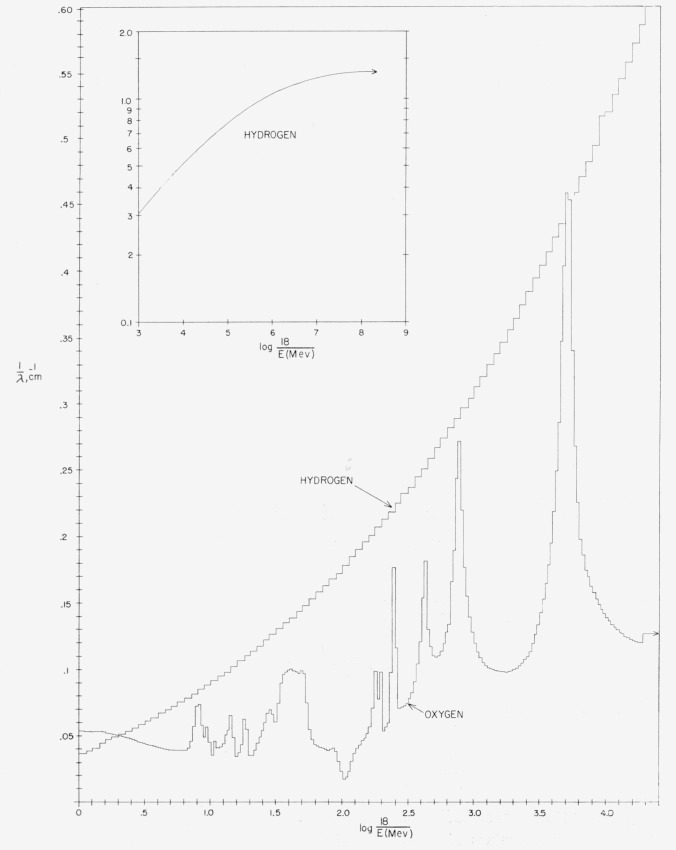
Attenuation coefficients for hydrogen and oxygen.

We have taken the values of *σ*_H_(*E*) and *σ*_Ox_(*E*) from the standard Brookhaven compilation [8]. The attenuation coefficients for hydrogen and oxygen thus obtained are shown separately in figure 2 as functions of the energy. The plots are made in histogram form corresponding to the approximate manner in which the data were used in the machine computations. It can be seen that the energy-dependence for hydrogen is smooth, but that the histogram for oxygen has several sharp peaks and irregularities at high energies.

Next we consider the relative contributions of various processes to the total collision cross section in the energy region of interest (0.5 ev to 14.0 Mev). For hydrogen, only elastic scattering is important in this region. The (*n,γ*) cross section is smaller than 0.1 percent of the total cross section at 0.5 ev, and is even less significant at higher energies.

For oxygen, the processes that matter are elastic scattering, inelastic scattering (above ≈ 6.4 Mev), and absorption due to (*n,α*) reactions (above ≈ 4 Mev) and (*n,p*) reactions (above ≈ 10 Mev). Cross sections for these processes, derived from the best experimental evidence and theoretical considerations, have been compiled by Lustig, Kalos, and Goldstein [9].^5^ In table 3.1 we list the values of the various cross sections at selected energies to indicate their relative importance. For use in machine calculations, we have fitted cross-section ratios with polynomials in the energy variable. The representations are a smoothed-out version of the data of reference [9] accurate to 5 percent or better, which is sufficient considering the uncertainty in the data.

**Table 3.1:** Neutron cross sections (barns) for oxygen (from reference [9])

Energy *Mev*	*^σ^*Ox *Total*	*^σ^α*, Ox *n, α*	*^σ^p*, Ox *n*, *p*	*^σ^n*′,Ox *Inelastic*

14	1.58	0.31	0.09	0.59
9	1.08	.25	……….	.21
6	1.48	.19	……….	……….
3	1.30	……….	……….	……….

$$\begin{array}{l} \text{Ratio of inelastic to total collision cross section}:\\ \frac{\sigma_{n\prime ,\mathrm{Ox}}}{\sigma_{\mathrm{Ox}}}=-1.05173+0.24196E-0.013666E^{2}+0.00024009E^{3}(6.4\;\mathrm{Mev}\leq E\leq 18\;\mathrm{Mev})\\ \text{Ratio of absorption to total collision cross section}:\\ \frac{\sigma_{\alpha ,\mathrm{Ox}}+\sigma_{p,\mathrm{Ox}}}{\sigma_{\mathrm{Ox}}}=-0.40433+0.13254E-0.0085614E^{2}+0.00017413E^{3}(4\;\mathrm{Mev}\leq E\leq 18\;\mathrm{Mev}) \end{array}\}$$ (3.3)

The probabilities that a given collision will be with a hydrogen or an oxygen nucleus are $\lambda_{H_{2}O}(E)/\lambda_{H}(E)$ and $\lambda_{H_{2}O}(E)/\lambda_{\mathrm{Ox}}(E)$, respectively. A collision can have four possible outcomes:

1. With probability
  $$p_{1}=\frac{\lambda_{H_{2}O}}{\lambda_{H}},$$ (3.4)
  the neutron is scattered elastically by a hydrogen nucleus.
2. With probability
  $$p_{2}=\frac{\lambda_{H_{2}O}}{\lambda_{\mathrm{Ox}}}\frac{\sigma_{\mathrm{Ox}}-\sigma_{n^{\prime },\mathrm{Ox}}-\sigma_{\alpha ,\mathrm{Ox}}-\sigma_{p,\mathrm{Ox}}}{\sigma_{\mathrm{Ox}}},$$ (3.5)
  the neutron is scattered elastically by an oxygen nucleus.
3. With probability
  $$p_{3}=\frac{\lambda_{H_{2}O}}{\lambda_{\mathrm{Ox}}}\frac{\sigma_{n^{\prime },\mathrm{Ox}}}{\sigma_{\mathrm{Ox}}},$$ (3.6)
  the neutron is scattered inelastically by an oxygen nucleus.
4. With probability
  $$p_{4}=\frac{\lambda_{H_{2}O}}{\lambda_{\mathrm{Ox}}}\frac{\sigma_{\alpha ,\mathrm{Ox}}+\sigma_{p,\mathrm{Ox}}}{\sigma_{\mathrm{Ox}}\;},$$ (3.7)
  the neutron is absorbed. Note that *p*_1_+*p*_2_+*p*_3_+*p*_4_=1.

### 3.2. Differential Cross Sections

The outcome of a collision in which a neutron of energy *E* is scattered can be specified in terms of the probability distribution *f_j_*(θ;*E*) of the scattering deflections θ*_c_* in the center-of-mass coordinate system. In conformity with the notation in the preceding subsection, we shall use indices *j*=1, 2, and 3 to indicate the distributions for elastic scattering from hydrogen, elastic scattering from oxygen, and inelastic scattering from oxygen, respectively.

For elastic scattering from hydrogen, the distribution is assumed to be isotropic,^6^ i.e.,

$$f_{1}(\theta_{c};E)=1.$$ (3.8)

**Figure 3:**
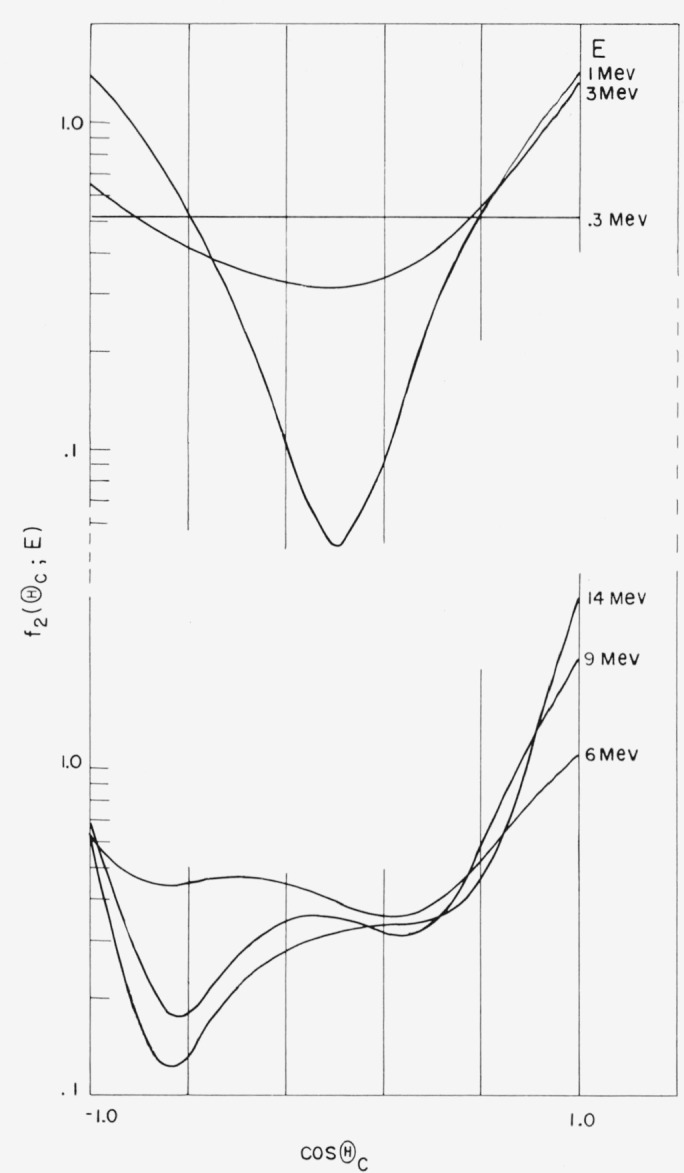
Angular distribution of elastic scattering from oxygen (according to ref. [9]).

The angular distribution for elastic scattering from oxygen is also isotropic at energies below about 300 kev, but markedly anisotropic at higher energies, particularly in resonance regions. Above 300 kev we have used the compilation of reference [9] which gives the coefficients *f*_2,_*_l_*(*E*) of the Legendre expansion

$$f_{2}(\theta_{c};E)=\sum_{l}\frac{2l+1}{2}f_{2,l}(E)P_{l}(\cos\;\theta_{c}).$$ (3.9)

(Note that the normalization is such that *f*_2,0_(*E*)=1.) Typical angular distributions at various energies are shown in figure 3.

The angular distribution for inelastic scattering from oxygen depends not only on the energy of the neutron before the collision but also on the excitation energy which the struck nucleus receives. In O^16^, the four lowest levels are 6.06, 6.14, 6.91, and 7.12 Mev above the ground state, the next is at 8.6 Mev and there are numerous levels between 9 and 14 Mev [11]. It appears from the discussion in reference [9] that neither the relative probabilities for the excitation of these levels nor the corresponding angular distributions are known. In order to explore the effect of inelastic scattering on the albedo, we have done three sets of calculations on the basis of the following assumptions:

1. The nucleus is excited to a level 6.1 Mev above the ground state. The angular distribution is isotropic; i.e.,
  $$f_{3}(\theta_{c};E)=1.$$ (3.10)
2. The neutron loses all its energy in an inelastic scattering, so that it is, in effect, absorbed.
3. An inelastic scattering is treated as if it were an elastic scattering; i.e., the excitation energy of the nucleus is set equal to zero.

The assumptions of case (1) seem to us to be the most realistic, and may be expected to yield reasonable answers when the neutron source energy is not too much above 9 Mev. The assumptions of case (2) lead to an underestimate of the albedo, and the assumptions of case (3) lead to an overestimate of the energy of the reflected neutrons.

## 4. Single-Scattering Albedo

### 4.1. Formulation

The probability that a neutron, incident on a semi-infinite medium with energy *E*_0_ and obliquity *θ*_0_, will be reflected with energy *E* and obliquity *θ* after exactly one collision is

$$\begin{array}{l} A^{\left(1\right)}(E,\theta ;E_{0},\theta_{0})=\int_{0}^{\infty }e^{-s/\lambda_{0}}\frac{ds}{\lambda_{0}}\sum_{j=1}^{3}p_{j}H_{j}(E,\theta ;E_{0},\theta_{0})e^{-s\prime /\lambda }\;\text{if}\;\pi /2<\theta \leq \pi ,\\ \;=0\;\text{if}\;0\leq \theta \leq \pi /2. \end{array}$$ (4.1)

In this expression, the factors $e^{-s/\lambda_{0}}\frac{ds}{\lambda_{0}}$ represents the probability that the incident neutron will travel a pathlength *s* and then undergo its first collision between *s* and *s+ds*, λ_0_=λ(*E*_0_) being the mean-free path. The collision can be of type 1 (elastic scattering from hydrogen), type 2 (elastic scattering from oxygen), or type 3 (inelastic scattering from oxygen). The fourth type (absorption) need not be considered. The factor *p_j_* (defined by (3.4, 3.5, and 3.6)) represents the probability that the collision will be of type *j.* The function *H_j_*(*E,θ;E*_0_,*θ*_0_) is called the *scattering function* and denotes the probability that in a collision of type *j* the energy of the neutron will change from *E*_0_ to *E* and the obliquity from *θ*_0_ to *θ.* Finally, exp(*−s*′/λ) is the probability that the neutron will travel from the point of collision to the boundary without making any more collisions (λ is the mean free path at the energy *E*). The pathlength to the boundary is

$$s\prime =-s\frac{\cos\;\theta_{0}}{\cos\;\theta }.$$ (4.2)

Using this relation, and carrying out the integration with respect to *8* in (4.1), we obtain the equation

$$\begin{array}{l} A^{\left(1\right)}(E,\theta ,E_{0},\theta_{0})=\sum_{j=1}^{3}\frac{p_{j}(E_{0})H_{j}(E,\theta ;E_{0},\theta_{0})\;\cos\;\theta }{\cos\;\theta -(\lambda_{0}/\lambda )\;\cos\;\theta_{0}}\;\text{if}\;\pi /2<\theta \leq \pi ,\\ \;=0\;\text{if}\;0\leq \theta \leq \pi /2. \end{array}$$ (4.3)

### 4.2. Scattering Function

The function *H_j_*(*E,θ;E*_0_,*θ*_0_) to be inserted into (4.3) can be derived from the corresponding distribution *f_j_*(θ_c_;*E*_0_) for the deflections in the center-of-mass coordinate system. To establish the connection, we need the relations between θ_c_, the corresponding deflection angle θ in the laboratory coordinate system, and the ratio of energies before and after the collision, *E*_0_/*E.* These relations are a consequence of the conservation of momentum and energy in the collision of a neutron with a nucleus. We state them for the case of inelastic scattering, characterized by the transfer of an amount *Q_j_* of energy to the target nucleus as excitation.^7^ The same relations also apply to elastic scattering if we set *Q_j_*=0.

The energy of the neutron after the collision is given by

$$E=E_{0}\frac{1+2M_{j}K_{j}\;\cos\;\theta_{c}+M_{j}^{2}K_{j}^{2}}{1+2M_{j}+M_{j}^{2}},$$ (4.4)

and the deflection in the laboratory system is

$$\cos\;\theta =\sqrt{\frac{E_{0}}{E}}\frac{1+M_{j}K_{j}\;\cos\;\theta_{c}}{1+M_{j}}=\frac{M_{j}+1}{2}\sqrt{\frac{E}{E_{0}}}-\frac{M_{j}^{2}K_{j}^{2}-1}{2(M_{j}+1)}\sqrt{\frac{E_{0}}{E}},$$ (4.5)

where M*_j_* is the mass of the target nucleus and where

$$K_{j}=\sqrt{1-\frac{Q_{j}}{E_{0}}\frac{1+M_{j}}{M_{j}}.}$$ (4.6)

Let *F_j_*(*E;E*_0_)*dE* denote the probability that the energy of the neutron after the collision lies between *E* and *E+dE.* Using (4.4) we have

$$F_{j}(E;E_{0})=f_{j}(\theta_{c};E_{0})\;\sin\;\theta_{c}\frac{d\theta_{c}}{dE}=\frac{{\left(M_{j}+1\right)}^{2}}{2M_{j}K_{j}E_{0}}f_{j}(\theta_{c};E_{0}).$$ (4.7)

According to (4.5), the deflection θ is determined by the value of *E*/*E*_0_. The joint distribution of *E* and θ can, therefore, be expressed formally with the use of a Dirac delta function, as

$$F_{j}(E;E_{0})\;\delta \;\left(\cos\;\theta -\frac{M_{j}+1}{2}\sqrt{\frac{E}{E_{0}}}+\frac{M_{j}^{2}K_{j}^{2}-1}{2(M_{j}+1)}\sqrt{\frac{E_{0}}{E}}\right).$$ (4.8)

Let the direction of the neutron before the collision be represented by a unit vector (sin*θ*_0_ cos *φ*_0_, sin*θ*_0_ sin*φ*_0_, cos*θ*_0_), and its direction after the collision by a unit vector (sin*θ* cos*φ*, sin*θ* sin*φ*, cos*θ*). Then the cosine of the deflection angle is the inner product of the two vectors,^8^

$$\cos\theta =\cos\theta_{0}\;\cos\theta +\sin\theta_{0}\;\sin\theta \;\cos(\varphi_{0}-\varphi ).$$ (4.9)

(The angles *φ*_0_ and *φ* are azimuths with respect to the *x–z* plane.) If we insert (4.9) into (4.8), introduce the abbreviation

$$\cos\;\alpha_{j}\equiv \frac{M_{j}+1}{2}\sqrt{\frac{E}{E_{0}}}-\frac{M_{j}^{2}K_{j}^{2}-1}{2(M_{j}+1)}\sqrt{\frac{E_{0}}{E}},$$ (4.10)

and carry out an azimuthal average with respect to *φ*′ = *φ*_0_−*φ*, we obtain the scattering function

$$\begin{array}{l} H_{j}(E,\theta ;E_{0},\theta_{0})=F_{j}(E,E_{0})\frac{1}{\pi }\int_{0}^{\pi }d\varphi \prime \delta (\cos\;\theta_{0}\;\cos\;\theta +\sin\;\theta_{0}\;\sin\;\theta \cos\;\varphi \prime -\cos\;\alpha_{j})\\ \;=\frac{F_{j}(E;E_{0})}{\pi {\left\{\cos(\alpha_{j}-\theta_{0})-\cos\;\theta \right\}}^{\frac{1}{2}}{\left\{\cos\;\theta -\cos(\alpha_{j}+\theta_{0})\right\}}^{\frac{1}{2}}}. \end{array}$$ (4.11)

The square roots in (4.11) must not become imaginary, which requires that

$$\cos(\alpha_{j}+\theta_{0})\leq \cos\theta \leq \cos(\alpha_{j}-\theta_{0}).$$ (4.12)

In the case of perpendicular incidence (*θ*_0_=0), the region (4.12) shrinks to a single value cos *α_j_* and the scattering function becomes

$$H_{j}(E,\theta ;E_{0},0)=F_{j}(E;E_{0})\delta (\cos\;\theta -\cos\;\alpha_{j}).$$ (4.13)

In the limiting case of grazing incidence (*θ*_0_ = 90°), the scattering function becomes

$$H_{j}(E,\theta ;E_{0},\pi /2)=\frac{F_{j}(E;E_{0})}{\pi \sqrt{\sin^{2}\theta -\cos^{2}\alpha_{j}}}.$$ (4.14)

### 4.3. Spectra and Angular Distributions

When using the single-scattering results as a guide for interpreting the Monte Carlo results, it is useful to have the differential albedo with one of the variables integrated out; i.e., the *energy spectrum*

$$A^{\left(1\right)}(E;E_{0},\theta_{0})=\int \sin\theta d\theta A^{\left(1\right)}(E,\theta ;E_{0},\theta_{0}),$$ (4.15)

and the *angular distribution*

$$A^{\left(1\right)}(\theta ;E_{0},\theta_{0})=\int dEA^{\left(1\right)}(E,\theta ;E_{0},\theta_{0}).$$ (4.16)

In the evaluation of these expressions the range of integration must include all values of *θ* for given *E* (or of *E* for given *θ*) for which the condition (4.12) is satisfied. Moreover, cos*θ* must be negative. The resultant formulas are somewhat complex. We give them in appendix A, confining ourselves here to some qualitative remarks.

The chief characteristic of the energy spectrum is that it is confined to limits which are wide for the case of hydrogen but very narrow for somewhat heavier nuclei such as oxygen. The upper limit of the spectrum follows from the condition that the neutron must be deflected sufficiently to acquire a velocity component in the negative *z*-direction. This implies, as a consequence of (4.12), that *E* cannot exceed

$$E_{\max}=E_{0}{\left\{\frac{\sqrt{M_{j}^{2}K_{j}^{2}-\cos^{2}\theta_{0}}+\sin\theta_{0}}{M_{j}+1}\right\}}^{2}.$$ (4.17)

The lower limit of the spectrum is determined by the conservation of momentum and energy which implies, according to (4.4), that *E* cannot be smaller than

$$E_{\max}=E_{0}{\left(\frac{M_{j}K_{j}-1}{M_{j}+1}\right)}^{2}.$$ (4.18)

**Figure 4:**
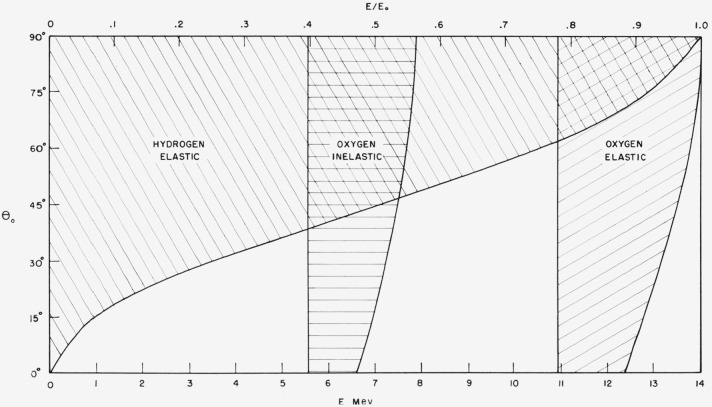
Possible energies which a neutron may have if it is reflected after a single scattering. The allowed energy regions for elastic scattering hold for all E_0_, whereas the indicated region for inelastic scattering applies only to E_0_=14 Mev and 6.1-Mev excitation.

The allowed energy regions between *E*_max_ and *E*_min_ are indicated in figure 4 as functions of the obliquity of incidence *θ*_0_.

The angular distribution cannot be described by any *one* simple law. For perpendicular incidence, the distribution follows approximately a cosine law; i.e., it peaks in directions corresponding to perpendicular emergence from the medium. As the direction of incidence becomes more oblique, this peak tends to disappear. In the limiting case of grazing incidence the angular distribution becomes flat or may even peak in directions corresponding to emergence parallel to the boundary of the semi-infinite medium. The angular distribution under these circumstances also becomes a sensitive function of the distribution *f*(θ*_c_*;*E*_0_) of scattering deflections. For details, see appendix A.

## 5. Monte Carlo Method

### 5.1. Generalities

Although the general principles of the Monte Carlo method are well known,^9^ we give a brief review to make this paper self-contained. The usual assumption is made that a neutron undergoing multiple scattering travels in a straight line until it makes a collision with a nucleus, is then suddenly deflected, travels again in a straight line till the next collision, and so on. The trajectory of a neutron is, therefore, completely described by a specification of the initial conditions and of the energies, obliquities, and positions of the neutron immediately after successive collisions. In the physical process, these characteristics are chance variables whose probability distributions are determined by the neutron cross sections. The Monte Carlo method consists of imitating nature by playing a game of chance. With the use of random numbers, the parameters of model neutron trajectories are calculated according to the appropriate probability distributions. Such a procedure is called random sampling, and a neutron trajectory thus generated is usually called a *neutron history.*

By examining a sufficiently large number of neutron histories, one can obtain statistical estimates of various transport phenomena. For example, if *N*_0_ histories are generated in each of which the neutron starts at the boundary of a semi-infinite medium, and if in *N* of these histories the neutron eventually emerges from the medium, then the ratio *N*/*N*_0_ is an estimate of the number albedo *A_N_.*

### 5.2. Random Sampling of Neutron Histories

If we let *E_n_*, *θ_n_*, and *z_n_* represent the energy, obliquity, and distance from the boundary immediately after the *n*’th collision, the neutron trajectory or history can be described by the array

$$\begin{array}{l} E_{0},\;E_{1},\;\ldots ,\;E_{n},\;\ldots \\ \theta_{0},\;\theta_{1},\;\ldots ,\;\theta_{n},\;\ldots \\ z_{0},\;z_{1},\;\ldots ,\;z_{n},\;\ldots \end{array}$$ (5.1)

in which *E*_0_, *θ*_0_, and *z*_0_=0 indicate the initial conditions. The history ends when the neutron is absorbed or reflected from the semi-infinite medium. We have adopted an additional termination rule in the present work, ending a history when the energy of the neutron drops below 0.5 ev. In order to generate a history, one must proceed from collision to collision, computing (*E_n_*_+1_,*θ_n_*_+1_,*z_n_*_+1_) from (*E_n_*,*θ_n_*,*z_n_*) recursively by random sampling.

By “random sampling from a distribution *f*(*x*)” we mean, in principle, the following procedure. A set of numbers, *x*_1_, *x*_2_, …, is prepared whose frequency distribution is made to approximate *f*(*x*) as closely as possible. One of these numbers is then selected at random and is called a *random variate* from the distribution *f*(*x*). It is, in fact, sufficient to prepare only a single set of random variates, from a uniform distribution *f*(*x*) = 1(0*≤x≤*1). These numbers, distributed uniformly between zero and one, are called *random numbers.* When suitably manipulated they can be made to yield random variates from any distribution, as shown below.

In a machine calculation requiring many random numbers it would be awkward to prepare and store a large set of random numbers. Instead, it is customary to use so-called *pseudorandom numbers* which are generated systematically but have, nevertheless, sufficient randomness for Monte Carlo calculations. We have employed the method of congruential multiplication^10^ in order to produce a sequence of pseudorandom numbers

$$\xi_{1},\;\xi_{2},\;\ldots ,\;\xi_{n}\;\ldots .$$ (5.2)

These numbers are fractions (between zero and one) which are obtained by normalizing a set of integers *x_n_*,

$$\xi_{n}=2^{-35}x_{n}.$$ (5.3)

The integers, in turn, are obtained from the following recursive scheme:

$$\begin{array}{l} x_{0}=c\\ x_{n+1}=5^{k}x_{n}\mathrm{mod}2^{35}, \end{array}\}$$ (5.4)

where *c* and *k* are odd integers. The last equation states that *x_n_*_+1_ is congruent to *5^k^x_n_* modulo 2^35^; in other words, that *x_n_*_+l_ is the remainder obtained upon division of 5*^k^x_n_* by 2^35^. In the course of our Monte Carlo calculations we have used three pseudorandom number sequences of the type (5.2), with *k*= 11, 13, and 15. This will be discussed more fully in section 5.4.

It can be proved (see app. B) that the sequence (5.2) contains 2^33^ different numbers which arc distributed with uniform density between zero and one. The successive random numbers used in the Monte Carlo calculation have been obtained by picking *successive* numbers in the pseudorandom number sequence (5.2). This is equivalent to a random choice, because the pseudorandom number sequence closely resembles a true random number sequence in the sense that there is no noticeable correlation between successive numbers. This lack of correlation has not been proved, but is an empirical fact that can be demonstrated by statistical tests. In appendix B, section 9, we present the results of such tests for 237,000 pseudorandom numbers actually used in our calculations.

One procedure for selecting a random variate *x* from a distribution *f*(*x*) by the manipulation of random numbers depends on the possibility of finding a random variate *y*(*x*) which is distributed uniformly between 0 and 1. We let

$$y(x)=\int_{x}^{\infty }f(x\prime )dx\prime ,$$ (5.5)

i.e., *y*(*x*) is the cumulative probability distribution associated with *f*(*x*). Note that

$$\text{probability}\;\{y<y\prime \}=\text{probability}\;\{x>x(y\prime )\}=\int_{x(y\prime )}^{\infty }f(x\prime )dx\prime =y\prime ,$$ (5.6)

so that *y* is equally likely to have any value between 0 and 1. We therefore identify *y* with a random number *ξ*, and determine a random variate *x* by inverting the equation

$$\xi =\int_{x}^{\infty }f(x\prime )dx.$$ (5.7)

A second method, called the “rejection technique” applies to distributions with a finite range, say 0*≤x≤a.* Let *L* be the maximum of *f*(*x*). The sampling procedure consists of the following steps. First, two random numbers, say *ξ*_1_ and *ξ*_2_, are selected. If *Lξ*_2_*≤f*(*aξ*_1_) we chose the number *aξ*_1_ as a random variate from *f*(*x*). If *Lξ*_2_>*f*(*aξ*_1_) we reject the random numbers *ξ*_1_ and *ξ*_2_ and repeat the testing process with a new pair of random numbers. The justification for this selection procedure is supplied by the observation that

$$\text{probability}\{\text{acceptance of}\;x=a\xi_{1}\}=\text{probability}\;\{L\xi_{2}\geq f(a\xi_{1})\}=\int_{0}^{f(a\xi_{1})/L}d\xi_{2}=\frac{f(x)}{L}.$$ (5.8)

The average number of random number pairs that must be tested before finding an acceptable random variate is given by

$$\frac{1}{\frac{1}{L}\int_{0}^{1}f(a\xi_{1})d\xi_{1}}=aL.$$ (5.9)

We shall now describe the actual sampling procedure for generating a neutron history; i.e., the rule for going from (*E_n_*,*θ_n_*,*z_n_*) to (*E_n_*_+1_,*θ_n_*_+1_,*z_n_*_+1_).

#### a. Location of the Next Collision

The probability distribution of *z_n_*_+1_ is

$$\frac{1}{\lambda (E_{n})}e^{-s_{n+1}/\lambda (E_{n})}=\frac{1}{\lambda (E_{n})}e^{-\frac{z_{n+1}-z_{n}}{\cos\;\theta_{n}}\frac{1}{\lambda (E_{n})}},$$ (5.10)

where

$$s_{n+1}=\frac{z_{n+1}-z_{n}}{\cos\;\theta_{n}}$$ (5.11)

is the pathlength traveled by the neutron between the *n*th and *n* + 1st collision. The distribution (5.10) is derived from the assumption that the spatial distribution of target nuclei is random and that the probability of a collision per unit pathlength is 1/λ(*E_n_*). Applying the cumulative-probability method, we select a random number *ξ_α_*, and determine *s_n_*_+1_ from the equation

$$\xi_{\alpha }=\int_{s_{n+1}/\lambda (E_{n})}^{\infty }e^{-s/\lambda (E_{n})}\frac{ds}{\lambda (E_{n})}=e^{-s_{n+1}/\lambda (E_{n})}.$$ (5.12)

The pathlength determines the position of the next collision, which is given by

$$z_{n+1}=z_{n}+\cos\theta_{n}s_{n+1}=z_{n}-\cos\theta_{n}\lambda (E_{n})\;\log\;\xi_{\alpha }.$$ (5.13)

#### b. Type of Collision

The probabilities *p_j_*(*j* = 1, 2, 3, 4) for the occurrence of each of the four possible types of collisions are given by (3.4), (3.5), (3.6), and (3.7). To make a random selection, we divide the region between 0 and 1 into four intervals proportional to the probability for each process. A random number *ξ_β_* is then picked and the type of collision is determined by the interval into which *ξ_β_* falls:

$$\begin{array}{cc} 0\leq \xi_{\beta }\leq p_{1} & \text{: elastic scattering from hydrogen}\\ p_{1}\leq \xi_{\beta }\leq p_{1}+p_{2} & \text{: elastic scattering from oxygen}\\ p_{1}+p_{2}<\xi_{\beta }\leq p_{1}+p_{2}+p_{3} & \text{: inelastic scattering from oxygen}\\ p_{1}+p_{2}+p_{3}<\xi_{\beta }\leq p_{1}+p_{2}+p_{3}+p_{4}=1 & \text{: absorption}. \end{array}$$

#### c. Energy Loss

If the *n* + 1st collision results in a scattering, the energy of the neutron is changed from *E_n_* to *E_n+_*_1_ according to the single-scattering law (4.7); i.e., the probability distribution of *E_n+_*_1_ is

$$F_{j}(E_{n+1};E_{n})=\frac{{\left(M_{j}+1\right)}^{2}}{2M_{j}K_{j}E_{0}}f_{j}(\theta_{c,n+1};E_{n}),$$ (5.14)

where

$$\cos\;\theta_{c,n+1}=\frac{{\left(M_{j}+1\right)}^{2}E_{n+1}-(1+M_{j}^{2}K_{j}^{2})E_{n}}{2M_{j}K_{j}E_{n}}.$$ (5.15)

The choice of the energy loss can be made indirectly through the choice of the center-of-mass deflection θ*_c_*_,_ *_n_*_+1_. When the distribution of deflections is isotropic (for collisions of type 1 and 3), we set

$$\cos\;\theta_{c,n+1}=2\xi_{\gamma }-1,$$ (5.16)

where *ξ_γ_* is a random number. For elastic scattering from oxygen, the angular distribution is anisotropic and is given by the Legendre expansion (3.9). In this case it is convenient to use the rejection technique. Provisionally, a deflection angle is chosen according to (5.16). An additional random number, $\xi_{\gamma }^{\left(1\right)}$, is then selected and a test is made to determine if

$$f_{2}\{\cos^{-1}(2\xi_{\gamma }-1);E_{n}\}\geq L(E_{n})\xi_{\gamma }^{\left(1\right)},$$ (5.17)

where *L*(*E_n_*) is the largest possible value of *f*_2_(θ*_c_*;*E_n_*). If the inequality (5.17) is satisfied, the provisional random variate given by (5.16) is accepted; if not, the selection procedure is repeated with two new random numbers.^11^

#### d Change of Obliquity

The prescription for selecting the obliquity after the *n*+1st collision can be obtained in two different ways: by a direct geometrical argument, or by an application of the scattering function derived in section 4. Starting with the latter, we note that, according to (4.11) the probability distribution of *θ_n_*_+1_ (for fixed θ*_c_*_,_*_n_*_+1_ and *θ_n_*) is given by

$$\frac{1}{\pi {\left\{\cos(\alpha_{jn}-\theta_{n})-\cos\theta_{n+1}\right\}}^{\frac{1}{2}}{\left\{\cos\theta_{n+1}-\cos(\alpha_{jn}+\theta_{n})\right\}}^{\frac{1}{2}}},$$ (5.18)

where

$$\cos\;\alpha_{jn}=\frac{M_{j}+1}{2}\sqrt{\frac{E_{n+1}}{E_{n}}}-\frac{M_{j}^{2}K_{j}^{2}-1}{2(M_{j}+1)}\sqrt{\frac{E_{n}}{E_{n+1}}},$$ (5.19)

and

$$\cos(\alpha_{jn}+\theta_{n})\leq \cos\theta_{n+1}\leq \cos(\alpha_{jn}-\theta_{n}).$$ (5.20)

Applying the cumulative-probability method, we pick a random number, *ξ_δ_*, and set

$$\begin{array}{l} 1-\xi_{\delta }=\frac{1}{\pi }\int_{\theta_{n+1}}^{\alpha_{jn}+\theta_{n}}\frac{\sin\theta d\theta }{{\left\{\cos(\alpha_{jn}-\theta_{n})-\cos\theta \right\}}^{\frac{1}{2}}{\left\{\cos\theta -\cos(\alpha_{n}+\theta_{jn})\right\}}^{\frac{1}{2}}}\\ \;=1-\frac{1}{\pi }\cos^{-1}\left\{\frac{\cos\theta_{n+1}-\cos\alpha_{jn}\;\cos\theta_{n}}{\sin\alpha_{jn}\;\sin\theta_{n}}\right\}. \end{array}$$ (5.21)

The inversion of this equation yields the rule for calculating *θ_n_*_+1_:

$$\cos\theta_{n+1}=\cos\alpha_{jn}\;\cos\;\theta_{n}+\sin\alpha_{jn}\;\sin\theta_{n}\;\cos({\pi \xi }_{\delta }).$$ (5.22)

**Figure 5:**
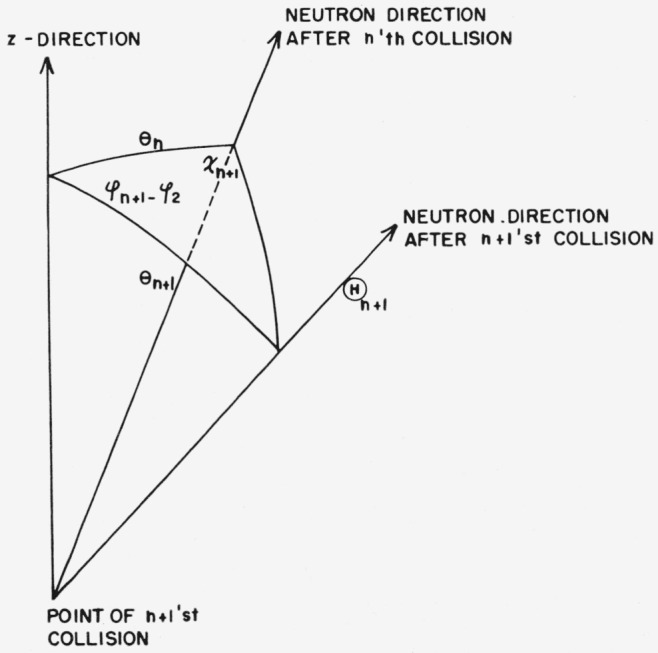
Spherical triangle illustrating the kinematics of a neutron scattering.

The geometrical derivation goes as follows. Let θ*_n_*_+1_ be the polar deflection and *χ_n_*_+1_ the associated azimuthal deflection in the laboratory system, resulting from the *n*+1st collision. *χ_n_*_+1_ is assumed to be measured with respect to a plane that contains the *z*-axis and the direction of motion of the neutron before the *n*+1st collision.) The various deflection angles and obliquities form a spherical triangle that is sketched in figure 5. The application of the law of cosines to this triangle yields the relation

$$\cos\theta_{n+1}=\cos\theta_{n+1}\;\cos\;\theta_{n}+\sin\theta_{n+1}\;\sin\theta_{n}\;\cos\chi_{n+1}.$$ (5.23)

Now, according to (4.5) and (4.10), θ*_n_*_+1_ is identical with *α_jn_.* Furthermore, it can be assumed that the azimuthal deflection angle *χ_n+_*_1_ is distributed randomly between 0 and 2*π*, so that it is legitimate to replace cos*χ_n_*_+1_ in (5.23) by cos*πξδ*, where *ξδ* is a random number. Thus (5.23) is in fact identical with (5.22).

### 5.3. Albedo Estimates

The estimate of the number albedo *A_N_* involves merely the determination of the fraction *N*/*N*_0_ of the number of histories that end in reflection.^12^ To obtain an expression for the statistical error of such an estimate we shall be somewhat formal and assign to each sampled history a “score” *R*, which has the value 1 in case of reflection and is 0 otherwise. Thus, *R* is a random variable which assumes the value 1 with probability *A_N_* and the value 0 with probability 1−*A_N_.*

The mean value of *R* is

$$\bar{R}=A_{N}\cdot 1+(1-A_{N})\cdot 0=A_{N}$$ (5.24)

and its variance (defined as $\bar{R^{2}}-{\bar{R}}^{2}$) is

$$\mathrm{var}\;R=A_{N}(1-A_{N}).$$ (5.25)

Suppose now that we sample and examine *N*_0_ histories with scores *R*_1_,*R*_2_, …, *R_n_*_0_. An estimate of the number albedo is given by the average score

$$A_{N}^{*}=\frac{1}{N_{0}}(R_{1}+R_{2}+\ldots +R_{N_{0}})=\frac{N}{N_{0}}.$$ (5.26)

The variance of this estimate^13^ is

$$\mathrm{var}\;A_{N}^{*}=\frac{1}{N_{0}}\mathrm{var}\;R=\frac{1}{N_{0}}A_{N}(1-A_{N}).$$ (5.27)

An estimate of the energy albedo can he obtained by multiplying the number albedo estimate *N*/*N*_0_ by the quantity $\bar{E}/E_{0}$, where $\bar{E}$ is the average energy of the reflected neutrons. More formally, we assign to each history a score *T* which is equal to *E*/*E*_0_ in case of reflect ion and which vanishes otherwise. The random variable *T* has the mean value

$$\bar{T}=A_{N}\frac{\bar{E}}{E_{0}},$$ (5.28)

and a variance

$$\mathrm{var}\;T=\bar{T^{2}}-\bar{T^{2}}=\frac{1}{E_{0}^{2}}\left(A_{N}\bar{E^{2}}-A_{N}^{2}{\bar{E}}^{2}\right).$$ (5.29)

An estimate of the energy albedo is provided by the average score

$$A_{E}^{*}=\frac{1}{N_{0}}(T_{1}+T_{2}+\ldots +T_{N_{0}})=\frac{N}{N_{0}}\frac{\bar{E}}{E_{0}}.$$ (5.30)

The variance of this estimate is

$$\begin{array}{c} \mathrm{var}\;A_{E}^{*}=\frac{1}{N_{0}E_{0}^{2}}\left(A_{N}\bar{E^{2}}-A_{N}^{2}{\bar{E}}^{2}\right)\\ =\frac{1}{N_{0}E_{0}^{2}}\left(\bar{E^{2}}\;\mathrm{var}\;R+A_{N}\;\mathrm{var}\;E\right). \end{array}$$ (5.31)

The two terms contributing to this variance are both non-negative. The first arises from fluctuations of the number of reflected neutrons and the second from variations of the energies of the reflected neutrons.

The significance of the variances var $A_{N}^{*}$ and var $A_{E}^{*}$ must be interpreted with reference to the probability distributions of $A_{N}^{*}$ and $A_{E}^{*}$. Both of these quantities are the averages of *N*_0_ identically distributed independent random variables. According to the central limit theorem of statistics [14] their distributions approach a Gaussian distribution in the limit of large *N*_0_.^14^ One can therefore make the following statements about the deviations of the albedo estimates from the true albedo values:

$$\text{Probability}\left\{\left|A_{N}^{*}-A_{N}\right|\geq k\sqrt{\mathrm{var}\;A_{N}^{*}}\right\}=\text{Probability}\left\{\left|A_{E}^{*}-A_{E}\right|\geq k\sqrt{\mathrm{var}\;A_{E}^{*}}\right\}=\sqrt{\frac{2}{\pi }}\int_{k}^{\infty }e^{-x^{2}/2}dx.$$ (5.32)

### 5.4. Correlated Random Sampling

To establish the dependence of the albedo on the assumed conditions (source energy and obliquity, collision cross sections, etc.), it is better to use a difference method than to make two or more separate calculations. The difference method can be designed to eliminate irrelevant statistical fluctuations, by keeping constant all random elements in the calculation which are not affected by the change of assumed conditions. Thereby greater accuracy can be achieved with a given amount of computation.

We recall from section 5.2. that the following random numbers specify the nature and outcome of a collision: *ξ_α_* determines the quantity *s_n_*_+1_/λ(*E_n_*) which is the distance (in mean free paths) traveled by the neutron between the previous collision and the collision under consideration; *ξ_β_* determines the type of collision; *ξ_γ_*, and, for anisotropic elastic scattering from oxygen, additional numbers $\xi_{\gamma }^{\left(1\right)}$, $\xi_{\gamma }^{\left(2\right)}$, … determines the center-of-mass deflection θ*_c_*_,_*_n_*_+1_; finally, *ξ_δ_* determines the azimuthal scattering deflection *χ_n+_*_1_. The above characteristics, together with the initial conditions, specify a history completely.

Suppose now that we want to make a direct determination of the difference in the albedo brought about by a change of the obliquity of the incident radiation. We can do this by generating simultaneously two *correlated sets of histories*, one set with one initial obliquity, the second set with another. The sets should be correlated in the sense that for each history in one set there exists a counterpart in the second set in which exactly the same random numbers are used for determining the outcomes of corresponding collisions. Thus two matched histories are alike, except that one of them is “rotated with respect to the other” and may be shorter because of the different initial obliquity. We call this case one of *maximal correlation*.^15^

In order to find out how a change in the assumed cross sections affects the albedo, we may again generate two (or more) correlated sets of histories, using identical random numbers for corresponding choices. Then two matched histories will at least be identical in regard to the distances (in mean free paths) between successive collisions, and in regard to successive azimuthal deflections. However, energy losses in corresponding collisions may be different. Consequently all the parameters of a history which depend on the energy may also be different. We call this case one of *partial correlation.* Even in this case, the use of identical random numbers tends to minimize accidental differences.

It is most economical, but not absolutely necessary, to generate several correlated sets of histories simultaneously. In the present work we wanted to compare the albedo under many different conditions, and used the neutron histories also for the solution of other problems, so that simultaneous computation of all correlated histories was not practicable. We therefore had to devise a technique for doing separate but correlated calculations. This was facilitated by the use of pseudorandom number sequences, which according to (5.3) and (5.4), are completely determined by a “starting value” *c* and a “multiplier” 5*^k^*, and can be conveniently regenerated provided we remember these two parameters.

The number of different random numbers required to generate two histories is usually not the same, even when they are correlated. In the first place, even in the case of maximal correlation, the two histories will tend to differ in length. Secondly, in the case of partial correlation there is a statistical variability in the number of random numbers $\xi_{\gamma }^{\left(1\right)}$, $\xi_{\gamma }^{\left(2\right)}$, … that may be required to select a scattering deflection by the rejection technique, according to (5.17). Thus it could happen that in spite of the use of the same pseudorandom number sequence (with the same *c* and *k*), different portions of this sequence would be used for supposedly correlated histories. This difficulty was circumvented by the use of many sequences of pseudorandom numbers instead of just one. A primary sequence was introduced with an arbitrary odd starting value *c* and a multiplier $5^{k_{1}}$. Successive members of the primary sequence were then used as the starting values of secondary sequences generated with a multiplier $5^{k_{2}}$. Successive secondary sequences were used for the calculation of successive histories. When the need arose for random sampling by the rejection technique, the requisite random numbers $\xi_{\gamma }^{\left(1\right)}$, $\xi_{\gamma }^{\left(2\right)}$, … were obtained from a tertiary sequence. The tertiary sequence used a multiplier $5^{k_{3}}$, and a starting value equal to the last member of the secondary sequence currently being used.^16^

We now discuss the effect of correlated sampling on Monte Carlo estimates of $A_{N}-{\tilde{A}}_{N}$ and $A_{E}-{\tilde{A}}_{E}$, where *A_N_* and *A_E_* are albedos under condition 1, and ${\tilde{A}}_{N}$ and ${\tilde{A}}_{E}$ the albedos under condition 2. Proceeding as in section 5.3, we assign to histories generated under condition 1 the scores *R* and *T*, and to the histories generated under condition 2 the scores $\tilde{R}$ and $\tilde{T}$. Albedo differences can then be estimated as follows:

$$A_{N}^{*}-{\tilde{A}}_{N}^{*}=\frac{1}{N_{0}}\{(R_{1}-{\tilde{R}}_{1})+(R_{2}-{\tilde{R}}_{2})+\ldots +(R_{N_{0}}-{\tilde{R}}_{N_{0}})\},$$ (5.33)

$$A_{E}^{*}-{\tilde{A}}_{E}^{*}=\frac{1}{N_{0}}\{(T_{1}-{\tilde{T}}_{1})+(T_{2}-{\tilde{T}}_{2})+\ldots +(T_{N_{0}}-{\tilde{T}}_{N_{0}})\}.$$ (5.34)

The variances of these difference estimates are

$$\mathrm{var}\;(A_{N}^{*}-{\tilde{A}}_{N}^{*})=\frac{1}{N_{0}}\{\mathrm{var}\;R-2\rho (R,\tilde{R})\sqrt{\mathrm{var}\;R\;\mathrm{var}\;\tilde{R}}+\;\mathrm{var}\;\tilde{R}\},$$ (5.35)

$$\mathrm{var}\;(A_{E}^{*}-{\tilde{A}}_{E}^{*})=\frac{1}{N_{0}}\{\;\mathrm{var}\;T-2\rho_{E}(T,\tilde{T})\sqrt{\mathrm{var}\;T\;\mathrm{var}\;\tilde{T}}+\;\mathrm{var}\;\tilde{T}\}.$$ (5.36)

The quantity

$$\rho (R,\tilde{R})=\frac{\bar{R\tilde{R}}-\bar{R}\cdot \bar{\tilde{R}}}{\sqrt{\mathrm{var}\;R\;\mathrm{var}\;\tilde{R}}}$$ (5.37)

is the correlation coefficient of R and $\tilde{R}$. Similarly,

$$\rho_{E}(T,\tilde{T})=\frac{\bar{T\tilde{T}}-\bar{T}\cdot \bar{\tilde{T}}}{\sqrt{\mathrm{var}\;T\;\mathrm{var}\;\tilde{T}}}$$ (5.38)

is the correlation coefficient of *T* and $\tilde{T}$. For uncorrelated pairs of histories, the correlation coefficients vanish. The objective of correlated sampling is to reduce var $\left(A_{N}^{*}-{\tilde{A}}_{N}^{*}\right)$ and var $\left(A_{E}^{*}-{\tilde{A}}_{E}^{*}\right)$ by making the correlation coefficients positive and as large as possible.

The magnitude of the actual correlation coefficients must, in general, be estimated from the sampled data, and some typical values will be given in section 7. It is possible, however, to put a theoretical upper limit on $\rho (R,\tilde{R})$. Using (5.25) and (5.37) we find that

$$\rho (R,\tilde{R})=\frac{\bar{R\tilde{R}}-A_{N}{\tilde{A}}_{N}}{\sqrt{A_{N}(1-A_{N}){\tilde{A}}_{N}(1-{\tilde{A}}_{N})}}.$$ (5.39)

Without loss of generality we may assume that ${\tilde{A}}_{N}\leq A_{N}$.

Therefore

$$\text{probability}\{R=1\;\text{and}\;\tilde{R}=1\}\leq \text{probability}\{\tilde{R}=1\}={\tilde{A}}_{N},$$ (5.40)

so that

$$\bar{R\tilde{R}}\leq {\tilde{A}}_{N}.$$ (5.41)

Accordingly the correlation coefficient must obey the inequality

$$\rho (R,\tilde{R})\leq \sqrt{\frac{{\tilde{A}}_{N}(1-A_{N})}{A_{N}(1-{\tilde{A}}_{N})}}\leq 1.$$ (5.42)

The greatest possible value of $\rho (R,\tilde{R})$ corresponds to the case of maximal correlation discussed above. It leads to a reduction of var $\left(A_{N}^{*}-{\tilde{A}}_{N}^{*}\right)$ by a factor

$$1-\frac{2}{\frac{A_{N}}{{\tilde{A}}_{N}}+\frac{1-{\tilde{A}}_{N}}{1-A_{N}}}.$$ (5.43)

## 6. Computations and Results

### 6.1. Machine Computations

The Monte Carlo calculations were programed for the IBM-704 computer at the National Bureau of Standards.^17^ The neutron histories, generated in order to determine the albedo, were concurrently used for the calculation of the neutron flux in infinite media and in slabs,^18^ so that it is difficult to state how much computer memory and computing time were required for the albedo problem alone. Altogether, a little more than 5,000 words of magnetic core memory were required of which not less than one-third were allocated to the albedo problem. For each case treated, 3,000 neutron histories were generated and analyzed. This amount of computation gave the desired accuracy and required approximately 45 min of computer time. Not more than half of this time was required by the albedo problem. The use of magnetic tapes was not necessary, but was an optional device for storing the results of one machine run and combining them with the results of a later run. Intermediate print-outs of the results were made after groups of 1,000 histories had been processed, in order to follow the course of the computations and to have a check on the performance of the computer.

In line with the exploratory nature of the investigation, the machine program was kept flexible. This made possible the exploration of new aspects of the problem as they emerged in the course of the work. In the simplest version of the program only elastic scattering from hydrogen and oxygen was taken into account, and the print-out of results was limited to tables of the differential albedo. In the latest version all relevant collision cross sections were included, and much additional information was printed out that had been found necessary for an understanding of the physical and statistical significance of the Monte Carlo results. This additional information included the mean and mean square energy of the reflected neutrons, the contributions of different orders of scattering to the number albedo and energy albedo, listings of the particular histories in which reflection occurred and of the energies of the reflected neutrons, and a statistical analysis of the pseudorandom numbers used. Intermediate versions of the program yielded only some of this information.

The analytical formulas for the single-scattering albedo, developed in section 4, were used mainly for the interpolation of results, and single-scattering computations were done with the Monte Carlo code. This was simpler to do once the machine program was available but slightly less accurate.

Altogether 38 different cases were treated which are listed in table 6.1. This table contains the source energy and obliquity as well as a brief indication of the assumptions about the cross sections. The computations fall into two groups: 30 cases were done with one set of pseudorandom numbers (analyzed in app. B), and the remaining 8 cases with another set. A high degree of correlation was introduced by the repeated use of the same set of pseudorandom numbers in order to increase the accuracy of intercomparisons. We believed this to be a sound procedure, because the set was large enough (consisting of approximately 237,000 numbers) to be statistically representative.

**Table 6.1:** Summary of calculations The entry in the left column serves as index to the differential-albedo tables in appendix C. Each result for the number albedo *A_N_* and the energy albedo *A_E_* is based on the analysis of 3,000 neutron histories, with *δA_N_* and *δA_E_* indicating the statistical standard deviations. All runs were done with the same set of pseudorandom numbers (described in app. B), except the runs indicated by an asterisk (*) which were all done with another set of pseudorandom numbers.

Table	*E*_0_(Mev)	*θ*_0_	*A_N_*	*δA_N_*	*A_E_*	*δA_E_*	Assumptions

1	0.3	0°	0.296	0.008	0.048	……….	(*).
None	1	0°	.370	.009	(.135)	……….	(*).
2a	1	0°	.361	.009	(.115)	……….	(*); elastic scattering from oxygen assumed isotropic.
2b	1	0°	.381	.009	.148	0.005	
2c	1	0°	.113	.006	.091	……….	Single scattering.
3a	1	45°	.450	.009	.159	……….	
3b	1	45°	.114	.006	.085	……….	Single scattering.
4a	1	60°	.518	.009	.183	……….	
4b	1	60°	.142	.006	.099	……….	Single scattering.
5a	1	75°	.619	.009	.241	……….	
5b	1	75°	.213	.007	.144	……….	Single scattering.
6a	1	90°	.767	.008	.415	……….	
6b	1	90°	.493	.009	.347	……….	Single scattering.
7a	3	0°	.061	.004	.023	……….	2-cm slab; see section 7.9; (*).
7b	3	0°	.131	.006	.038	……….	5-cm slab; see section 7.9; (*).
7c	3	0°	.163	.007	.044	……….	10-cm slab; see section 7.9; (*).
None	3	0°	.168	.007	(.044)	……….	(*).
8a	3	0°	.178	.007	(.049)	……….	(*); elastic scattering from oxygen assumed isotropic.
8b	3	0°	.167	.007	.041	.003	
8c	3	0°	.028	.003	.023	……….	Single scattering.
9a	3	45°	.232	.008	.052	……….	
9b	3	45°	.035	.003	.023	……….	Single scattering.
10a	3	60°	.315	.008	(.080)	……….	
10b	3	60°	.066	.005	(.039)	……….	Single scattering.
11a	3	75°	.472	.009	.144	……….	
11b	3	75°	.143	.006	.084	……….	Single scattering.
12a	3	90°	.728	.008	(.339)	……….	
12b	3	90°	.480	.009	(.290)	……….	Single scattering.
13	3	(†)	.371	.009	(.110)	……….	
14a	6	0°	.157	.007	.052	.003	
14b	6	0°	.171	.007	.059	.004	Absorption disregarded.
15a	9	0°	.107	.006	.027	.002	Excitation of 6.1-Mev level in oxygen by inelastic scattering.
15b	9	0°	.138	.006	.041	.003	Absorption disregarded.
15c	9	0°	.131	.006	.047	……….	Absorption disregarded. Inelastic scattering from oxygen treated as elastic.
16a	14	0°	.124	.006	.040	.003	Excitation of 6.1-Mev level in oxygen by inelastic scattering.
16b	14	0°	.163	.007	.038	.002	Elastic scattering from oxygen assumed isotropic; excitation of 6.1-Mev level in oxygen by inelastic scattering.
16c	14	0°	.165	.007	.079	.004	Absorption disregarded. Inelastic scattering from oxygen treated as elastic.
16d	14	0°	.064	.004	.023	.002	Inelastic scattering treated as absorption.

(†) Isotropic source.

### 6.2. Differential Albedo

Tables 1 to 16d of appendix C contain the Monte Carlo results for the differential albedo in the form in which they were printed out by the computer. The number of reflected neutrons (for *N*_0_=3,000 neutrons) is given in a two-way classification according to the spectral energy, *E*, and the cosine of the obliquity of emergence, cos *θ.* By normalizing the entries in the tables, i.e., by dividing through by *N*_0_, one obtains an estimate of the differential albedo averaged over a small energy-angle interval. Unnormalized numbers are given in the tables in order to preserve their statistical significance, the standard deviation of each entry being proportional to its square root. The energy classification is divided into 10 intervals, labeled *i*=1, 2, … 10, such that the *i*th interval pertains to neutrons with energies between (11− *i*)*/*10*E*_0_ and (10−*i*)/10*E*_0_. The last interval extends from *E*_0_/10 to 0.5 ev rather than to zero energy. The obliquity classification is also divided into 10 intervals, labeled *k*= 1, 2,…, 10, such that the *k*th interval pertains to neutrons with obliquity cosines between (1*−k*)*/*10 and *−k/*10. Two sets of marginal totals are also given which represent the angular distribution integrated over all energies, and the spectral distribution integrated overall directions, respectively.

### 6.3. Number Albedo and Energy Albedo

Table 6.1 contains all the results obtained for the number albedo *A_N_* and the energy albedo *A_E_*.^19^ The number albedo was obtained by adding the marginal totals in the corresponding differential-albedo tables and dividing the result *by N_0_*=3,000. The energy albedo was obtained as the product of the number albedo and the average energy *E* of the reflected neutrons. In most of the calculations *A_E_* was computed directly in the course of the machine calculations. In some of the earlier cases, $\bar{E}$ had to be estimated from the differential-albedo tables.^20^ Numerical experimentation indicated that such estimates could be made with an accuracy of approximately 1 percent. Values of the energy albedo based on such estimates of *E* are indicated in table 6.1. by being placed in parentheses.

The standard deviations $\delta A_{N}=\sqrt{\mathrm{var}\;A_{N}}$ and $\delta A_{E}=\sqrt{\mathrm{var}\;A_{E}}$ are also shown in table 6.1. They were computed according to (5.27) and (5.31), respectively. The determination of *δA_E_* required knowledge of $\bar{E}$ as well as of ${\bar{E}}^{2}$. Accordingly, *δA_E_* is given only for those cases in which ${\bar{E}}^{2}$ was obtained as direct output of the machine program. The relative standard deviation *δA_N_/A_N_*, obtained with 3,000 histories, is of the order of 0.05. The corresponding relative standard deviation *δA_E_/A_E_*, in the cases examined and presumably also in the other cases, is roughly 1½ times greater than *δA_N_/A_N_.*

## 7. Analysis of the Results

### 7.1 Single-Scattering Contribution to the Albedo

Table 7.1 contains a summary of the ratios $A_{N}^{\left(1\right)}/A_{N}$ and $A_{E}^{\left(1\right)}/A_{E}$ for various cases. In general, single scattering contributes about 20 percent of the number albedo and more than 50 percent of the energy albedo. The contributions are particularly large at 1 Mev, because at this energy the ratio of the scattering cross section of oxygen to that of hydrogen is large, and the differential oxygen scattering cross section has a peak in the backward direction.

The estimation, of the albedo ratios is a problem with maximal correlation as defined in section 5.4. We have estimated the standard deviations of these ratios in a few typical cases and find, for example, that $A_{N}^{\left(1\right)}/A_{N}=0.30\pm 0.03$ and $A_{E}^{\left(1\right)}/A_{E}=0.61\pm 0.02$ for *E*_0_= 1 Mev and *θ*_0_=0°.

The ratio $A_{E}^{\left(1\right)}/A_{E}$ is a relatively slowly varying function of the source energy, whereas $A_{E}^{\left(1\right)}$ and *A_E_* separately vary rapidly. This suggests that the energy albedo for arbitrary energies could be obtained with fair accuracy by combining a single-scattering calculation with an interpolated value of $A_{E}^{\left(1\right)}/A_{E}$.

**Table 7.1:** Ratio of single-scattering albedo to total albedo. (The results for 6, 9, and 14 Mev correspond to cases 14a, 15a, and 16a, respectively, as listed in table 6.1).

$\frac{{A_{N}}^{\left(1\right)}}{A_{N}}$						$\frac{{A_{E}}^{\left(1\right)}}{A_{E}}$					
*θ*_0_	0°	45°	60°	*75°*	90°	*θ*_0_	0°	45°	60°	75°	90°
*E*_0_ (Mev)						*E*_0_ (Mev)					

0.3	0.11					0.3	0.54				
1	.30	0.25	0.27	0.34	0.64	1	.61	0.54	0.54	0.60	0.84
3	.17	.15	.21	.30	.66	3	.55	.45	.48	.58	.85
6	.22					6	.54				
9	.17					9	.42				
14	.27					14	.50				

### 7.2. Contribution of Higher Orders of Scattering

**Figure 6:**
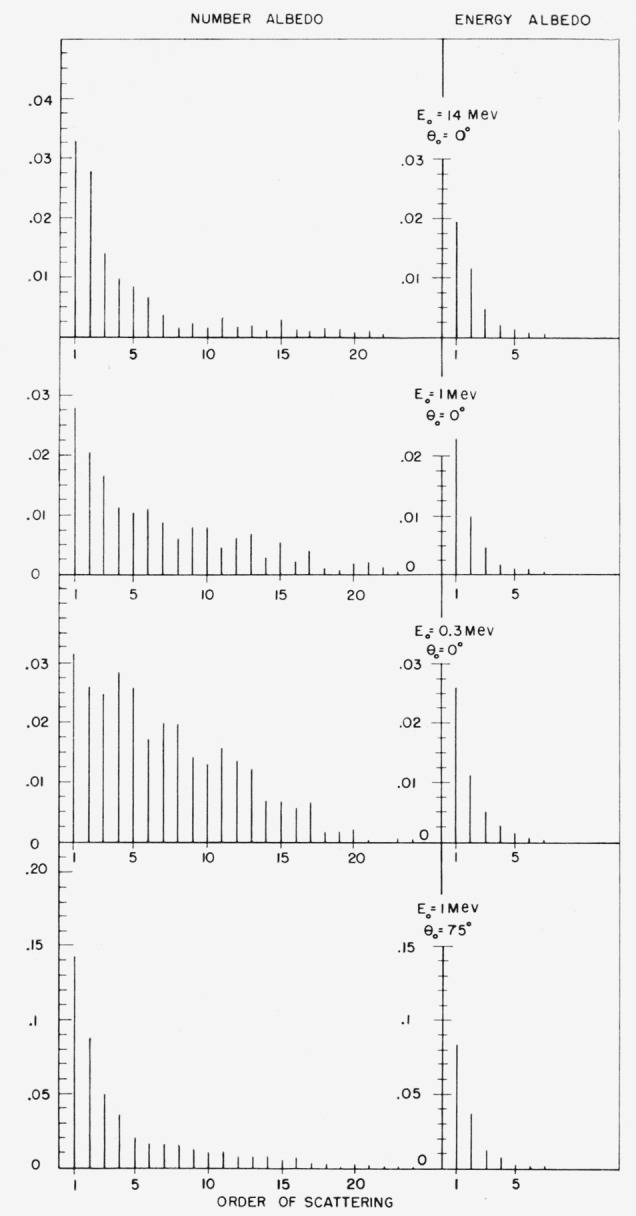
Contributions of successive orders of scattering to the albedo.

Figure 6 shows the contributions of successive orders of scattering to the albedo for a few typical cases. As many as twenty orders are shown to contribute to the number albedo,^21^ whereas only about five orders contribute significantly to the energy albedo. The relative importance of the first few orders of scattering increases with the source obliquity and energy. In general, an analytical orders-of-scattering approach does not seem to provide a practical alternative to a Monte Carlo calculation. The contribution of the second order of scattering is not sufficient for a precise albedo determination, and calculations for the higher orders would be prohibitively complex.

### 7.3. Energy Spectra of Reflected Neutrons

**Figure 7:**
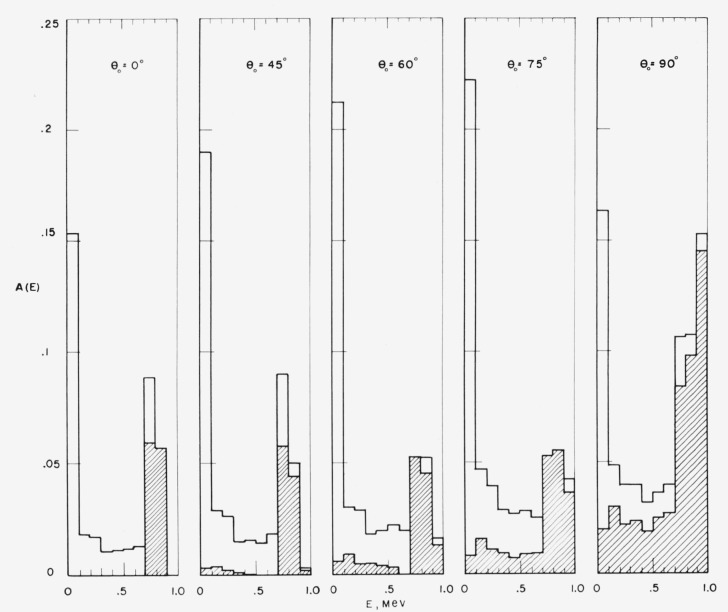
Energy spectra of reflected neutrons. The shaded areas indicate the contribution of single-scattering. Source energy 1 Mev.

Figure 7 contains histograms of the energy distribution of reflected neutrons for incident 1-Mev beams with various obliquities. The contributions of single scattering and multiple scattering to the spectra are indicated. The spectra are characterized by two rather sharp peaks: one, primarily due to single scattering, occurs at the upper end of the spectra; the other, due to multiple scattering, at the low-energy end. The single-scattering contribution can in turn be resolved into two components, due to scattering from oxygen and hydrogen. For perpendicular incidence, only single-scattering reflection from oxygen occurs. As the source obliquity is increased, hydrogen scattering becomes possible, and the oxygen contribution is shifted toward higher energies. At 45° and 60° the oxygen and hydrogen components are clearly separated, whereas at 75° and 90° they merge. In the limiting case of grazing incidence there is a hydrogen component distributed uniformly over all energies, and an oxygen component which gives rise to a peak at an energy slightly below the source energy. Further details about the oxygen single-scattering contribution can be obtained from appendix B (fig. 15). The energy spectra for other cases are similar to those shown in figure 7 and can be obtained from the tables in appendix C.

### 7.4. Angular Distribution of Reflected Energy

**Figure 8:**
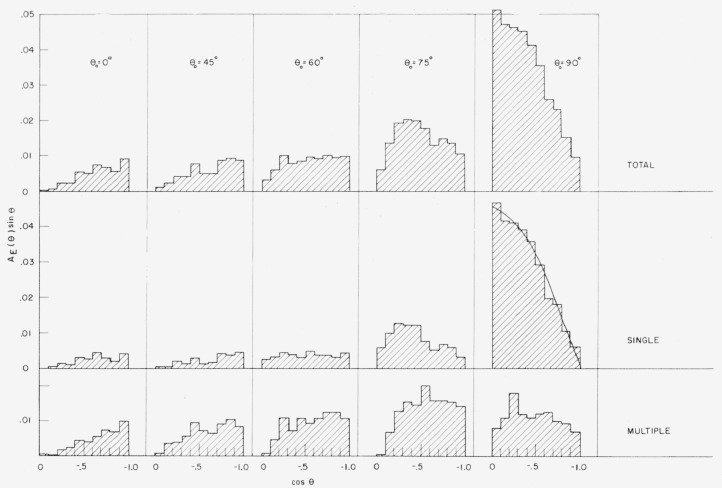
Angular distribution of reflected energy. Source energy 3 Mev. A single-scattering curve for *θ*_0_=90° is shown which corresponds to eq (7.2).

Figure 8 shows plots, in histogram form, of the quantity

$$A_{E}(\theta ;E_{0},\theta_{0})=\int_{0}^{E_{0}}A(E,\theta ;E_{0},\theta_{0})\frac{E}{E_{0}}dE,$$ (7.1)

for *E*_0_=3 Mev and various values of *θ*_0_.^22^ The angular distribution is dominated by the singlescattering contribution. In particular, it should be noted that for large source obliquities the distribution does not follow the cosine-law which holds for perpendicular incidence and has often been assumed to be universally valid. Rather the emerging radiation tends to peak for cos*θ*=0; i.e., in directions parallel to the boundary of the semi-infinite medium. This is due to the behavior of the single-scattering component. For *E*_0_=3 Mev and *θ*_0_=90°, we find from eq (A.21), (A.22), and (A.24) of appendix A, and from the cross sections given in figure 2, that

$$A_{E}^{\left(1\right)}\sim 0.049(1.00+1.05\;\sin^{2}\theta +7.14\;\sin^{3}\theta ),$$ (7.2)

which is in agreement with the Monte Carlo calculations.

### 7.5. Dependence of Albedo on Source Energy and Obliquity

The dependence on the source energy is irregular. It is correlated with the complicated energy-dependence of the attenuation coefficient for water, and is further influenced by the relative probabilities for scattering by hydrogen and oxygen, and by the energy-dependent anisotropy of elastic scattering from oxygen. Just like the attenuation coefficient, the number albedo and energy albedo have a peak at 1 Mev where they are 2 to 3 times larger than at the other energies considered. The correlation between attenuation coefficients and albedo is complicated, however, by the interaction of the various factors, so that interpolation with respect to source energy is not easy, and is perhaps done best by the single-scattering analysis indicated in section 7.1.^23^

**Figure 9:**
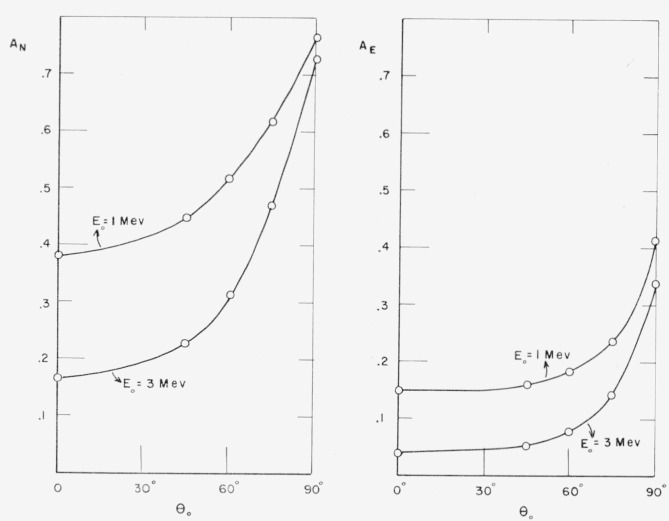
Dependence of albedo on source obliquity.

The dependence on the source obliquity is smooth and interpolation should be easy. By way of example, figure 9 contains curves of *A_N_* and *A_E_* as functions of *θ*_0_, for source energies of 1 and 3 Mev. All four curves shown have similar shapes, starting out with a rather flat slope near *θ*_0_=0° and rising very steeply near *θ*_0_=90°. The closer *θ*_0_ is to grazing incidence, the smaller are the differences of the albedos for different source energies.

### 7.6. Effect of Absorption and Inelastic Scattering

**Figure 10:**
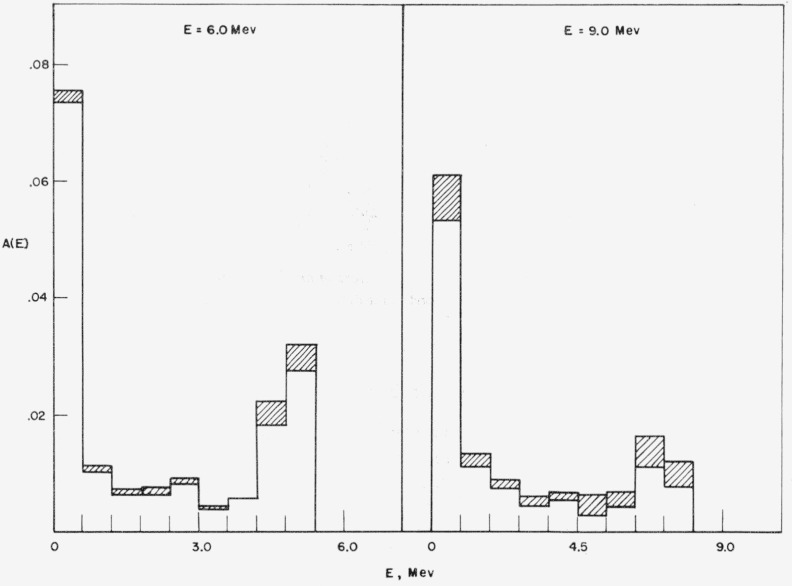
Reduction of energy spectrum due to absorption, indicated by shaded areas. Perpendicular incidence.

If absorption were disregarded, the number albedo at 6 Mev would be increased by 9 percent and the energy albedo by 13 percent. At 9 Mev, the corresponding increases would be 29 percent and 52 percent, respectively. The effect of absorption is greater on the energy albedo than on the number albedo because the relative probability of absorption is an increasing function of the energy (up to 14 Mev). The effect of absorption on the energy spectrum is shown in figure 10. Although the effect is large, it introduces no large uncertainty into the calculations because the cross section for *n* − *α* and *n* − *p* processes are reasonably well-known.

The effect of inelastic scattering is more difficult to determine because so little is known about the cross section for this process. We have made computations under three different- assumptions: (1) Absorption taken into account, inelastic scattering assumed to excite oxygen to a level 6.1 Mev above the ground state; (2) absorption taken into account, inelastic scattering also treated as absorption; (3) absorption disregarded, inelastic scattering treated as if it were elastic.

As has been discussed in section 3.2, assumption (1) is presumably the most realistic. Assumption (3) is the least realistic and has been included mainly because it has often been made in previous neutron transport calculations. Assumptions (1) and (2) are limiting assumptions in the sense that they allow for the minimum and maximum possible energy loss that a neutron could suffer in an inelastic collision. The albedo calculated on assumption (3) is almost twice as great as that calculated on the realistic assumption (1), whereas the albedo calculated on assumption (2) is only about half as large. These extreme variations point up the need for accurate knowledge of the inelastic scattering cross section.

**Figure 11:**
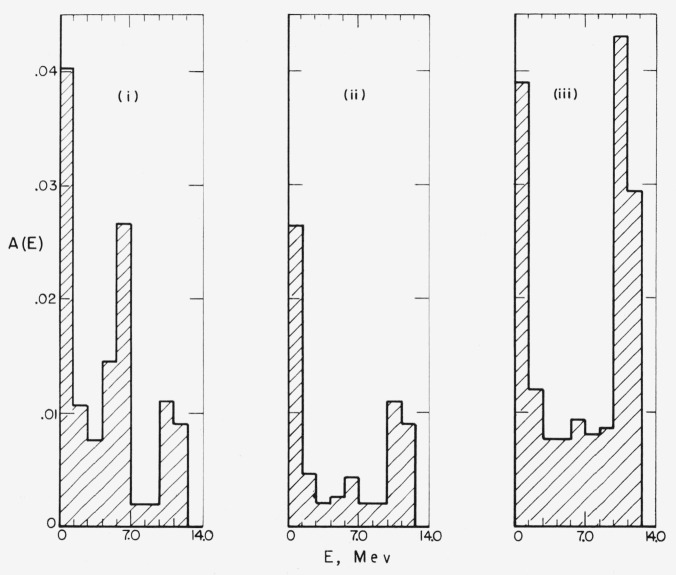
Effect of inelastic scattering on the energy spectrum. Source energy 14 Mev. Histograms (i), (ii), and (iii) correspond to the different assumptions in sec. 7.6, and were obtained from tables 16a 16d, and 16c of app. C, respectively. Perpendicular incidence.

The energy spectra obtained with a 14-Mev source under the three assumptions are shown in figure 11. The spectrum in case (1) shows three peaks, due to elastic single scattering, inelastic single scattering, and multiple scattering. The inelastic scattering peak disappears in case (2) and merges with the peak due to elastic single scattering in case (3). More accurate treatment of inelastic scattering would probably give a spectrum similar to that of case (1), but with the middle peak reduced in size and “smeared out” over the lower part of the spectrum.

### 7.7. Effect of the Anisotropy of Elastic Scattering

**Figure 12:**
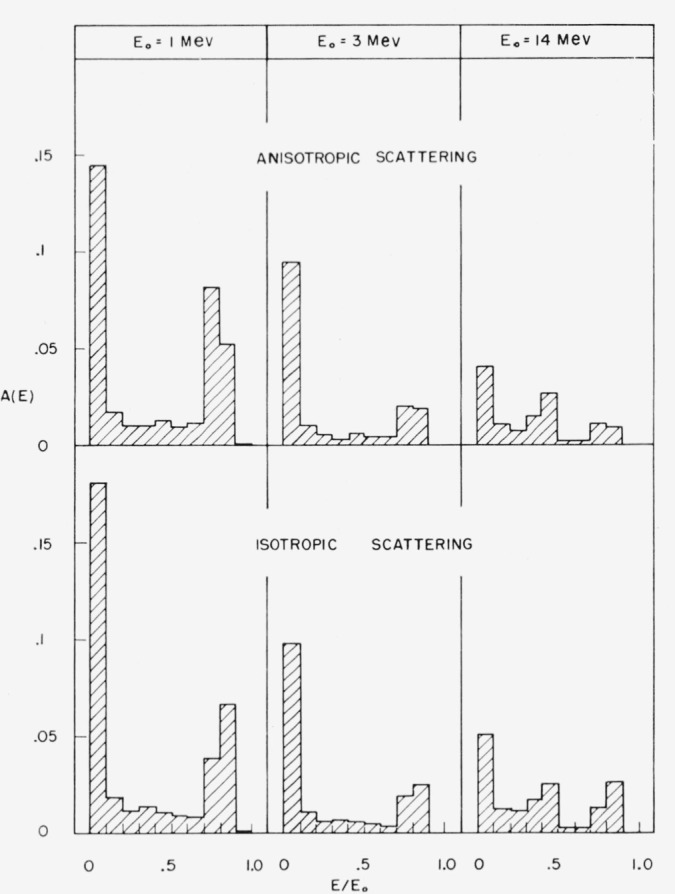
Effect of anisotropy of elastic scattering from oxygen on the energy spectrum. Perpendicular incidence.

The differential elastic scattering cross section is not well-known for most elements. It is therefore of interest to know how much the albedo would be changed by a change of the assumed distribution of elastic scattering deflections. To explore this situation, we have compared the albedo calculated with the best available cross sections for oxygen (reference [9], see also fig. 3) with the albedo calculated on the assumption that the elastic scattering deflections from oxygen are isotropic in the center-of-mass system. The difference between the two assumptions is greater than any expected realistic modification of the oxygen cross sections. Comparisons were made for source energies at 1, 3, and 14 Mev. At 1 Mev, *A_N_* is decreased by 2.5 percent and *A_E_* by 11 percent. Finally, at 14 Mev, *A_N_* is increased by 31 percent, whereas *A_E_* is decreased by 5 percent. In figure 12 the spectra obtained under the two different assumptions are compared. There is relatively little change, the main effect being a shift of the singlescattering peak toward higher energies in the case of isotropic scattering.

The albedo differences quoted above were obtained by correlated sampling (case of partial correlation). A listing of all significant statistical parameters for the comparison at 14 Mev is given in table 7.2 from which it appears that by means of correlated sampling an accuracy was achieved that would have required a sample size 3 to 4 times as large with ordinary sampling.

**Table 7.2:** Estimate of albedo difference by correlated sampling Source energy 14 Mev, perpendicular incidence. Elastic scattering from oxygen assumed to be anisotropic in case 16a, isotropic in case 16b.

*Number albedo:*	*Energy albedo:*
${\tilde{A}}_{N}$, case 16a,	${\tilde{A}}_{E}$, case 16a,
*A_N_*, case 16b,	*A_E_*, case 16b,
$A_{N}-{\tilde{A}}_{N}=0.039\pm 0.005$ (with correlated sampling), ±0.009 (without correlated sampling),	$A_{E}-{\tilde{A}}_{E}=-0.0012\pm 0.0016$ (with correlated sampling), ±0.0033 (without correlated sampling),
Correlated sampling increases effective sample size by factor 2.9,	Correlated sampling increases effective sample size by factor 3.9,
Correlation coefficient *ρ*=0.664,	Correlation coefficient *ρE*=0.771.
Maximal correlation coefficient *ρ*_max_=0.853,	

### 7.8. Low-Energy End of the Spectrum

**Figure 13:**
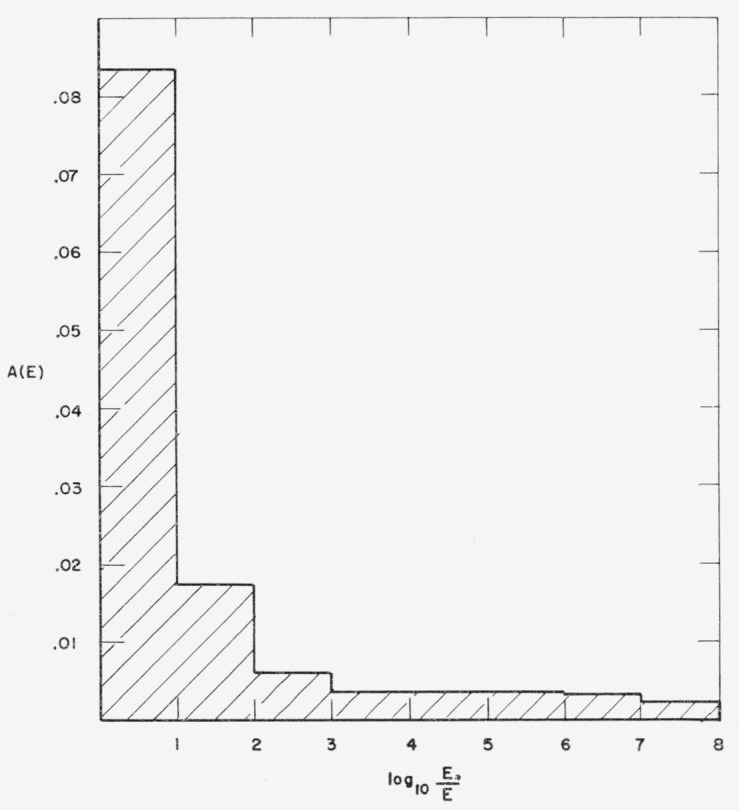
Energy spectrum of reflected neutrons on semilog scale. Source energy 14 Mev, perpendicular incidence.

Details of the low-energy part of the spectrum were obtained as a byproduct of the detailed information obtained for the statistical analysis of the correlated calculations at 14 Mev. Figure 13 shows a plot of the spectrum on a logarithmic scale which emphasizes the low energies. The spectral shape at low energies is quite similar to that obtained by Spinney [5] who calculated by age theory the albedo for 2-Mev neutrons incident on concrete. This suggests that computations of the spectrum down to very low energies could advantageously be done by a combination of Monte Carlo and age theory, with Monte Carlo covering the energy region not very far below the source energy in which age theory does not apply.

### 7.9. Slab Albedo

**Figure 14:**
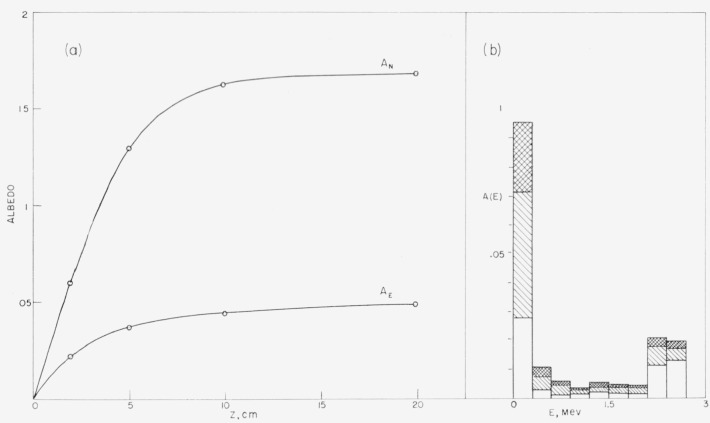
Dependence of slab albedo on slab thickness. Source energy 3 Mev, perpendicular incidence, a. Number and energy albedo and b. Energy spectrum.

**Figure 15:**
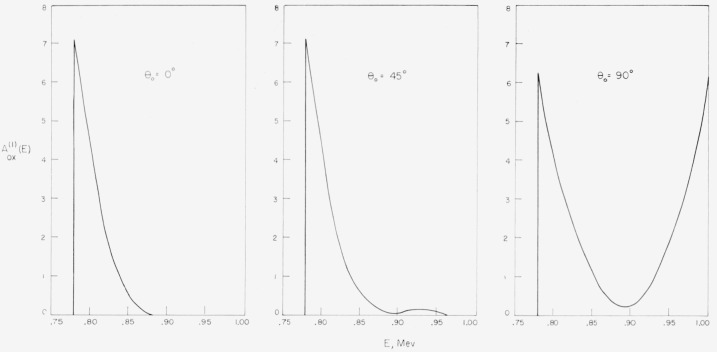
Energy spectrum of neutrons reflected after one collision. Source energy 1 Mev, oxygen medium.

The deeper a neutron penetrates into the medium, the smaller is its chance of returning. Therefore, the collisions leading to reflection take place rather close to the surface of the medium, and thin slabs have nearly the same reflecting power as a semi-infinite medium. This is shown by the results in figure 14 pertaining to the reflection of 3-Mev neutrons incident on slabs with thicknesses of 2, 5, 10, and 20 cm. (These thicknesses are to be compared with the transport mean-free path in water at 3 Mev which is 5.1 cm.) The dependence of the number albedo on the slab thickness is similar to that found by Foderaro and Obenshain [6] at 0.89 Mev. The energy albedo reaches a limiting value at an even smaller slab thickness than the number albedo, because the energy degradation in successive collisions makes it necessary for a neutron to be reflected very early in its history if it is to carry back an appreciable amount of energy.

## Figures and Tables

**Table 1 t1-jresv63an2p101_a1b:** 

cos *θ*												
	*k*	1	2	3	4	5	6	7	8	9	10	Sum
	*i*											
	
	1	……….	……….	……….	……….	……….	……….	……….	……….	……….	……….	0
	2	3	7	4	8	12	14	14	6	……….	……….	68
	3	1	2	1	3	1	5	1	3	15	18	50
	4	……….	……….	2	1	2	……….	1	2	3	3	14
*E*	5	……….	……….	……….	……….	……….	3	……….	3	1	3	10
	6	……….	……….	……….	……….	2	5	4	2	5	7	25
	7	……….	……….	……….	……….	1	4	5	2	3	3	18
	8	……….	……….	……….	1	2	1	3	2	2	2	13
	9	1	……….	1	2	4	5	6	6	4	6	35
	10	……….	10	16	49	48	75	91	101	124	142	656
	
	Sum	5	19	24	64	72	112	125	127	157	184	889

**Table 2a t2a-jresv63an2p101_a1b:** 

cos *θ*												
	*k*	1	2	3	4	5	6	7	8	9	10	Sum
	*i*											
	
	1	……….	……….	1	……….	……….	……….	……….	……….	……….	……….	1
	2	5	10	16	20	32	37	48	30	3	……….	201
	3	……….	……….	……….	7	4	7	4	14	40	41	117
	4	1	1	2	2	2	4	3	3	3	5	26
*E*	5	……….	2	1	1	5	3	5	4	2	5	28
	6	……….	1	……….	3	2	4	5	2	11	5	33
	7	……….	……….	2	1	5	9	8	3	9	6	43
	8	……….	1	2	1	1	5	5	7	3	10	35
	9	1	1	……….	3	5	4	6	15	11	10	56
	10	3	10	17	43	52	48	52	94	102	123	544
	
	Sum	10	26	41	81	108	121	136	172	184	205	1,084

**Table 2b t2b-jresv63an2p101_a1b:** 

cos *θ*												
	*k*	1	2	3	4	5	6	7	8	9	10	Sum
	*i*											
	
	1	……….	……….	……….	……….	……….	……….	……….	……….	……….	……….	0
	2	1	……….	5	10	21	32	46	56	1	……….	172
	3	1	2	2	5	6	5	12	34	86	113	266
	4	……….	……….	……….	2	5	3	3	6.	8	12	39
*E*	5	……….	1	……….	……….	3	5	5	11	3	7	35
	6	……….	1	2	……….	1	4	4	8	7	7	34
	7	……….	1	……….	3	7	5	1	4	7	4	32
	8	……….	……….	3	8	5	10	2	5	8	11	52
	9	2	5	……….	4	4	5	11	5	9	10	55
	10	4	7	22	37	49	51	51	64	86	88	459
	
	Sum	8	17	34	69	101	120	135	193	215	252	1,144

**Table 2c t2c-jresv63an2p101_a1b:** 

cos *θ*												
	*k*	1	2	3	4	5	6	7	8	9	10	Sum
	*i*											
	
	1	……….	……….	……….	……….	……….	……….	……….	……….	……….	……….	0
	2	1	……….	4	9	19	31	42	56	……….	……….	162
	3	……….	……….	……….	……….	……….	……….	……….	21	68	88	177
	4	……….	……….	……….	……….	……….	……….	……….	……….	……….	……….	0
*E*	5	……….	……….	……….	……….	……….	……….	……….	……….	……….	……….	0
	6	……….	……….	……….	……….	……….	……….	……….	……….	……….	……….	0
	7	……….	……….	……….	……….	……….	……….	……….	……….	……….	……….	0
	8	……….	……….	……….	……….	……….	……….	……….	……….	……….	……….	0
	9	……….	……….	……….	……….	……….	……….	……….	……….	……….	……….	0
	10	……….	……….	……….	……….	……….	……….	……….	……….	……….	……….	0
	
	Sum	1	0	4	9	19	31	42	77	68	88	339

**Table 3a t3a-jresv63an2p101_a1b:** 

cos *θ*												
	*k*	1	2	3	4	5	6	7	8	9	10	Sum
	*i*											
	
	1	1	2	1	3	2	……….	……….	……….	……….	……….	9
	2	4	4	13	9	16	11	17	21	18	37	150
	3	1	11	14	18	32	21	33	49	47	45	271
	4	1	1	2	7	7	6	7	10	6	8	55
*E*	5	1	4	1	3	4	10	9	2	4	4	42
	6	1	……….	2	2	2	7	8	7	10	7	46
	7	1	10	2	3	5	2	5	3	9	4	44
	8	1	4	9	6	12	10	7	6	14	9	78
	9	2	5	9	7	3	9	10	12	10	19	86
	10	8	11	24	42	56	62	59	84	100	123	569
	
	Sum	21	52	77	100	139	138	155	194	218	256	1,350

**Table 3b t3b-jresv63an2p101_a1b:** 

cos *θ*												
	*k*	1	2	3	4	5	6	7	8	9	10	Sum
	*i*											
	
	1	1	2	1	2	……….	……….	……….	……….	……….	……….	6
	2	4	3	10	6	12	10	16	18	16	37	132
	3	……….	7	6	9	22	14	22	31	35	27	173
	4	……….	……….	……….	……….	……….	……….	……….	……….	……….	……….	0
*E*	5	……….	……….	……….	……….	……….	……….	……….	……….	……….	……….	0
	6	1	……….	……….	……….	……….	……….	……….	……….	……….	……….	1
	7	……….	4	……….	……….	……….	……….	……….	……….	……….	……….	4
	8	1	……….	5	……….	……….	……….	……….	……….	……….	……….	6
	9	……….	1	4	5	1	……….	……….	……….	……….	……….	11
	10	……….	……….	……….	2	6	1	……….	……….	……….	……….	9
	
	Sum	7	17	26	24	41	25	38	49	51	64	342

**Table 4a t4a-jresv63an2p101_a1b:** 

cos *θ*												
	*k*	1	2	3	4	5	6	7	8	9	10	Sum
	*i*											
	
	1	6	10	6	9	8	7	2	……….	1	……….	49
	2	5	8	7	18	10	15	16	13	22	44	158
	3	4	14	19	27	19	38	46	41	35	18	261
	4	2	4	9	4	6	6	9	6	3	9	58
*E*	5	1	10	8	6	11	10	5	8	2	5	66
	6	1	3	12	5	5	7	8	10	7	1	59
	7	2	2	4	12	7	6	4	9	5	4	55
	8	1	4	8	12	9	13	11	6	9	13	86
	9	1	9	12	3	19	17	13	12	15	19	120
	10	7	15	29	55	43	79	84	85	119	121	637
	
	Sum	30	79	114	151	137	198	198	190	218	234	1,549

**Table 4b t4b-jresv63an2p101_a1b:** 

cos *θ*												
	*k*	1	2	3	4	5	6	7	8	9	10	Sum
	*i*											
	
	1	6	9	5	7	6	5	……….	……….	……….	……….	38
	2	4	6	2	14	9	13	12	12	21	43	136
	3	3	8	13	21	9	25	27	28	21	3	158
	4	1	……….	……….	……….	……….	……….	……….	……….	……….	……….	1
*E*	5	1	5	3	……….	……….	……….	……….	……….	……….	……….	9
	6	1	2	7	1	……….	……….	……….	……….	……….	……….	11
	7	……….	2	3	7	3	……….	……….	……….	……….	……….	15
	8	1	2	2	2	3	4	……….	……….	……….	……….	14
	9	……….	5	1	1	7	11	2	……….	……….	……….	27
	10	……….	2	2	1	2	4	4	2	……….	……….	17
	
	Sum	17	41	38	54	39	62	45	42	42	46	426

**Table 5a t5a-jresv63an2p101_a1b:** 

cos *θ*												
	*k*	1	2	3	4	5	6	7	8	9	10	Sum
	*i*											
	
	1	6	24	26	21	18	12	11	7	1	1	127
	2	12	20	23	19	11	15	17	26	35	23	201
	3	10	22	37	41	41	38	34	31	10	10	274
	4	3	4	16	16	9	6	5	8	2	7	76
*E*	5	4	8	9	13	20	10	10	4	4	3	85
	6	……….	4	9	7	20	13	10	12	4	2	81
	7	1	5	8	9	11	18	16	7	8	3	86
	8	5	5	17	10	14	14	10	21	8	15	119
	9	2	4	18	12	12	7	27	22	25	12	141
	10	3	18	27	47	56	86	87	90	137	116	667
	
	Sum	46	114	190	195	212	219	227	228	234	192	1,857

**Table 5b t5b-jresv63an2p101_a1b:** 

cos *θ*												
	*k*	1	2	3	4	5	6	7	8	9	10	Sum
	*i*											
	
	1	6	21	23	20	16	9	9	5	……….	……….	109
	2	12	17	13	14	10	9	11	25	35	21	167
	3	8	16	27	22	25	27	18	17	……….	……….	160
	4	3	……….	13	11	……….	……….	……….	……….	……….	……….	27
*E*	5	2	2	4	6	12	……….	……….	……….	……….	……….	26
	6	……….	……….	1	3	10	6	……….	……….	……….	……….	20
	7	……….	3	1	3	6	9	5	……….	……….	……….	27
	8	2	2	6	2	6	4	6	5	……….	……….	33
	9	2	3	6	3	2	2	15	10	4	……….	47
	10	……….	1	1	4	1	1	3	9	4	……….	24
	
	Sum	35	65	95	88	88	67	67	71	43	21	640

**Table 6a t6a-jresv63an2p101_a1b:** 

cos *θ*												
	*k*	1	2	3	4	5	6	7	8	9	10	Sum
	*i*											
	
	1	111	84	61	41	52	42	30	19	15	3	458
	2	45	33	41	56	20	30	35	30	24	8	322
	3	47	53	57	43	57	38	9	8	4	3	319
	4	11	13	18	14	20	24	5	4	7	4	120
*E*	5	11	11	18	15	15	13	22	2	1	1	109
	6	8	5	13	10	8	11	18	20	2	1	96
	7	4	11	15	11	9	17	18	26	8	1	120
	8	9	6	9	16	16	15	11	21	14	4	121
	9	7	6	11	14	17	10	13	21	30	17	146
	10	7	15	28	36	42	57	67	75	78	85	490
	
	Sum	260	237	271	256	256	257	228	226	183	127	2,301

**Table 6b t6b-jresv63an2p101_a1b:** 

cos *θ*												
	*k*	1	2	3	4	5	6	7	8	9	10	Sum
	*i*											
	
	1	107	80	58	39	48	39	29	18	14	3	435
	2	41	30	39	52	17	24	34	28	21	7	293
	3	44	46	47	37	48	29	1	……….	……….	……….	252
	4	9	11	12	8	18	21	2	……….	……….	……….	81
*E*	5	9	8	13	8	8	10	20	……….	……….	……….	76
	6	2	4	8	4	5	6	12	15	……….	……….	56
	7	3	7	9	5	6	10	8	20	3	……….	71
	8	7	3	6	9	7	7	5	11	11	……….	66
	9	6	5	7	6	8	4	8	16	19	11	90
	10	4	3	2	4	5	4	8	5	12	12	59
	
	Sum	232	197	201	172	170	154	127	113	80	33	1,479

**Table 7a t7a-jresv63an2p101_a1b:** 

cos *θ*												
	*k*	1	2	3	4	5	6	7	8	9	10	Sum
	*i*											
	
	1	……….	……….	……….	……….	……….	……….	……….	……….	……….	……….	0
	2	1	2	2	6	4	8	8	7	……….	……….	38
	3	……….	……….	……….	……….	……….	2	1	3	13	14	33
	4	……….	……….	……….	……….	2	……….	……….	2	……….	……….	4
*E*	5	……….	……….	……….	1	……….	……….	2	……….	……….	2	5
	6	……….	1	……….	1	……….	3	1	……….	……….	……….	6
	7	……….	1	……….	……….	……….	……….	1	1	1	……….	4
	8	……….	……….	……….	……….	1	……….	……….	1	1	……….	3
	9	……….	……….	……….	……….	2	1	……….	1	3	……….	7
	10	……….	5	1	4	7	7	13	19	13	14	83
	
	Sum	1	9	3	12	16	21	26	34	31	30	183

**Table 7b t7b-jresv63an2p101_a1b:** 

cos *θ*												
	*k*	1	2	3	4	5	6	7	8	9	10	Sum
	*i*											
	
	1	……….	……….	……….	……….	……….	……….	……….	……….	……….	……….	0
	2	1	2	2	8	5	11	14	9	……….	……….	52
	3	……….	……….	……….	1	……….	3	1	6	19	24	54
	4	……….	……….	……….	……….	3	1	……….	4	1	1	10
*E*	5	……….	……….	……….	1	……….	2	3	1	1	2	10
	6	……….	1	……….	2	1	3	2	……….	1	1	11
	7	……….	1	……….	……….	1	2	1	2	1	……….	8
	8	……….	……….	……….	……….	2	1	1	3	4	2	13
	9	……….	……….	1	……….	3	2	3	5	4	3	21
	10	1	8	6	10	13	18	29	42	38	48	213
	
	Sum	2	12	9	22	28	43	54	72	69	81	392

**Table 7c t7c-jresv63an2p101_a1b:** 

cos *θ*												
	*k*	1	2	3	4	5	6	7	8	9	10	Sum
	*i*											
	
	1	……….	……….	……….	……….	……….	……….	……….	……….	……….	……….	0
	2	1	2	2	8	7	12	14	11	……….	……….	57
	3	……….	……….	……….	1	……….	4	2	6	20	27	60
	4	……….	……….	……….	1	3	1	……….	5	1	2	13
*E*	5	……….	……….	……….	1	……….	2	3	2	1	3	12
	6	……….	1	……….	2	1	3	2	1	3	2	15
	7	……….	1	……….	……….	1	3	1	2	1	……….	9
	8	……….	……….	……….	1	2	2	1	3	5	3	17
	9	……….	……….	1	1	3	2	4	6	5	6	28
	10	1	9	9	12	15	24	41	51	51	64	277
	
	Sum	2	13	12	27	32	53	68	87	87	107	488

**Table 8a t8a-jresv63an2p101_a1b:** 

cos *θ*												
	*k*	1	2	3	4	5	6	7	8	9	10	Sum
	*i*											
	
	1	……….	……….	……….	……….	……….	……….	……….	……….	……….	……….	0
	2	2	5	5	7	13	18	15	10	……….	……….	75
	3	……….	1	……….	2	1	3	1	5	18	22	53
	4	……….	……….	2	2	1	……….	……….	2	2	2	11
*E*	5	……….	……….	……….	……….	1	3	1	3	3	3	14
	6	……….	1	……….	……….	2	2	5	1	5	2	18
	7	……….	……….	1	……….	……….	5	3	3	3	4	19
	8	……….	……….	……….	2	3	2	1	1	5	3	17
	9	……….	……….	1	2	2	3	8	6	7	5	34
	10	……….	10	11	17	23	27	49	50	48	59	294
	
	Sum	2	17	20	32	46	63	83	81	91	100	535

**Table 8b t8b-jresv63an2p101_a1b:** 

cos *θ*												
	*k*	1	2	3	4	5	6	7	8	9	10	Sum
	*i*											
	
	1	……….	……….	……….	……….	……….	……….	……….	……….	……….	……….	0
	2	……….	2	6	4	11	10	16	9	……….	1	59
	3	……….	……….	1	1	1	1	2	6	11	22	45
	4	1	……….	……….	2	1	2	2	2	1	3	14
*E*	5	……….	……….	……….	……….	2	2	3	4	1	1	13
	6	……….	……….	1	1	4	1	1	4	4	1	17
	7	……….	……….	……….	……….	1	1	2	1	4	3	12
	8	……….	……….	1	1	3	3	1	1	3	6	19
	9	……….	1	1	2	3	2	4	3	5	9	30
	10	1	5	10	16	22	30	49	41	53	66	293
	
	Sum	2	8	20	27	48	52	80	71	82	112	502

**Table 8c t8c-jresv63an2p101_a1b:** 

cos *θ*												
	*k*	1	2	3	4	5	6	7	8	9	10	Sum
	*i*											
	
	1	……….	……….	……….	……….	……….	……….	……….	……….	……….	……….	0
	2	……….	2	5	4	11	10	16	8	……….	……….	56
	3	……….	……….	……….	……….	……….	……….	……….	3	8	17	28
	4	……….	……….	……….	……….	……….	……….	……….	……….	……….	……….	0
*E*	5	……….	……….	……….	……….	……….	……….	……….	……….	……….	……….	0
	6	……….	……….	……….	……….	……….	……….	……….	……….	……….	……….	0
	7	……….	……….	……….	……….	……….	……….	……….	……….	……….	……….	0
	8	……….	……….	……….	……….	……….	……….	……….	……….	……….	……….	0
	9	……….	……….	……….	……….	……….	……….	……….	……….	……….	……….	0
	10	……….	……….	……….	……….	……….	……….	……….	……….	……….	……….	0
	
	Sum	0	2	5	4	11	10	16	11	8	17	84

**Table 9a t9a-jresv63an2p101_a1b:** 

cos *θ*												
	*k*	1	2	3	4	5	6	7	8	9	10	Sum
	*i*											
	
	1	1	1	2	1	2	……….	……….	……….	……….	……….	7
	2	……….	2	5	4	8	4	6	7	8	11	55
	3	……….	1	1	2	6	4	2	15	10	9	50
	4	1	……….	……….	2	5	……….	……….	3	1	3	15
*E*	5	1	3	1	2	1	4	2	……….	4	2	20
	6	2	……….	2	3	1	4	4	3	4	2	25
	7	……….	2	1	……….	3	1	4	3	6	2	22
	8	1	4	9	3	7	5	3	4	9	2	47
	9	1	2	5	7	9	8	7	10	6	11	66
	10	4	8	17	26	36	38	45	56	82	78	390
	
	Sum	11	23	43	50	78	68	73	101	130	120	697

**Table 9b t9b-jresv63an2p101_a1b:** 

cos *θ*												
	*k*	1	2	3	4	5	6	7	8	9	10	Sum
	*i*											
	
	1	1	1	2	1	2	……….	……….	……….	……….	……….	7
	2	……….	……….	3	2	6	2	5	6	8	11	43
	3	……….	……….	……….	1	2	3	1	10	7	6	30
	4	……….	……….	……….	……….	……….	……….	……….	……….	……….	……….	0
*E*	5	……….	……….	……….	……….	……….	……….	……….	……….	……….	……….	0
	6	1	……….	……….	……….	……….	……….	……….	……….	……….	……….	1
	7	……….	1	……….	……….	……….	……….	……….	……….	……….	……….	1
	8	1	1	6	……….	……….	……….	……….	……….	……….	……….	8
	9	……….	……….	3	2	1	……….	……….	……….	……….	……….	6
	10	……….	1	……….	2	6	……….	……….	……….	……….	……….	9
	
	Sum	3	4	14	8	17	5	6	16	15	17	105

**Table 10a t10a-jresv63an2p101_a1b:** 

cos *θ*												
	*k*	1	2	3	4	5	6	7	8	9	10	Sum
	*i*											
	
	1	3	6	5	5	4	6	3	1	……….	……….	33
	2	3	1	6	4	5	6	4	8	10	15	62
	3	2	1	5	5	4	6	11	12	7	5	58
	4	2	3	4	1	3	2	1	……….	4	3	23
*E*	5	……….	8	6	3	5	5	3	4	1	2	37
	6	2	2	14	5	3	5	5	5	3	2	46
	7	2	3	5	9	7	1	4	5	4	3	43
	8	1	5	8	8	12	13	10	9	9	5	80
	9	……….	3	2	6	10	10	9	10	17	16	83
	10	2	14	18	27	34	54	70	77	92	92	480
	
	Sum	17	46	73	73	87	108	120	131	147	143	945

**Table 10b t10b-jresv63an2p101_a1b:** 

cos *θ*												
	*k*	1	2	3	4	5	6	7	8	9	10	Sum
	*i*											
	
	1	3	5	5	3	4	6	2	1	……….	……….	29
	2	2	1	2	3	3	3	3	6	10	15	48
	3	2	……….	1	3	……….	6	9	7	2	1	31
	4	2	……….	……….	……….	……….	……….	……….	……….	……….	……….	2
*E*	5	……….	4	3	……….	……….	……….	……….	……….	……….	……….	7
	6	1	2	8	3	……….	……….	……….	……….	……….	……….	14
	7	1	2	2	7	2	……….	……….	……….	……….	……….	14
	8	1	3	3	3	5	4	……….	……….	……….	……….	19
	9	……….	2	……….	2	5	7	1	……….	……….	……….	17
	10	……….	1	……….	2	3	3	5	2	……….	……….	16
	
	Sum	12	20	24	26	22	29	20	16	12	16	197

**Table 11a t11a-jresv63an2p101_a1b:** 

cos *θ*												
	*k*	1	2	3	4	5	6	7	8	9	10	Sum
	*i*											
	
	1	5	9	12	14	10	10	5	6	5	……….	76
	2	10	13	10	10	7	9	5	12	18	13	107
	3	4	12	20	9	11	11	6	9	6	4	92
	4	2	2	12	22	6	4	2	2	1	3	56
*E*	5	1	4	4	10	22	6	7	3	3	2	62
	6	……….	11	7	7	22	13	10	5	4	4	83
	7	……….	5	9	8	7	19	10	9	5	5	77
	8	1	5	17	10	19	18	17	20	11	10	128
	9	3	2	9	14	7	14	31	29	14	19	142
	10	1	11	28	46	42	66	74	92	119	115	594
	
	Sum	27	74	128	150	153	170	167	187	186	175	1,417

**Table 11b t11b-jresv63an2p101_a1b:** 

cos *θ*												
	*k*	1	2	3	4	5	6	7	8	9	10	Sum
	*i*											
	
	1	5	9	11	13	9	7	5	6	4	……….	69
	2	10	10	3	3	6	3	3	12	16	12	78
	3	3	12	18	5	8	7	2	3	……….	……….	58
	4	2	1	10	18	……….	……….	……….	……….	……….	……….	31
*E*	5	1	2	4	5	13	……….	……….	……….	……….	……….	25
	6	……….	1	2	5	16	8	……….	……….	……….	……….	32
	7	……….	3	1	3	5	11	6	……….	……….	……….	29
	8	1	1	6	3	7	4	10	5	……….	……….	37
	9	1	2	4	4	1	4	13	7	1	……….	37
	10	……….	3	1	2	2	3	4	12	7	……….	34
	
	Sum	23	44	60	61	67	47	43	45	28	12	430

**Table 12a t12a-jresv63an2p101_a1b:** 

cos *θ*												
	*k*	1	2	3	4	5	6	7	8	9	10	Sum
	*i*											
	
	1	81	62	50	26	29	25	10	15	10	8	316
	2	38	30	38	57	19	13	16	16	14	11	252
	3	19	23	22	30	46	18	3	3	4	3	171
	4	17	20	19	16	26	38	7	……….	2	……….	145
*E*	5	16	20	19	22	17	20	29	2	1	1	147
	6	7	12	15	12	10	14	33	25	5	2	135
	7	7	17	19	12	15	22	17	36	7	2	154
	8	11	9	13	19	21	18	19	28	20	2	160
	9	13	10	16	11	20	11	23	28	38	18	188
	10	4	19	23	38	44	52	73	71	93	99	516
	
	Sum	213	222	234	243	247	231	230	224	194	146	2,184

**Table 12b t12b-jresv63an2p101_a1b:** 

cos *θ*												
	*k*	1	2	3	4	5	6	7	8	9	10	Sum
	*i*											
	
	1	77	60	50	26	27	25	10	15	10	8	308
	2	34	27	35	53	18	8	13	14	13	10	225
	3	18	20	20	29	45	17	1	……….	……….	……….	150
	4	15	17	15	15	25	37	5	……….	……….	……….	129
*E*	5	14	17	16	14	11	15	29	……….	……….	……….	116
	6	6	9	10	6	7	9	25	21	……….	……….	93
	7	5	10	15	9	10	14	8	31	5	……….	107
	8	9	3	8	8	10	10	9	19	17	……….	93
	9	11	8	10	6	9	6	14	20	28	11	123
	10	2	6	4	8	6	7	12	9	20	21	95
	
	Sum	191	177	183	174	168	148	126	129	93	50	1,439

**Table 13 t13-jresv63an2p101_a1b:** 

cos *θ*												
	*k*	1	2	3	4	5	6	7	8	9	10	Sum
	*i*											
	
	1	5	15	6	8	5	6	5	2	1	3	56
	2	6	5	7	13	5	10	10	6	12	21	95
	3	5	10	9	11	9	5	9	6	5	10	79
	4	1	3	8	3	6	6	2	3	……….	1	33
*E*	5	2	7	3	5	9	4	4	2	2	4	42
	6	2	6	7	6	5	14	12	8	6	4	70
	7	1	3	8	7	11	10	10	9	6	7	72
	8	2	7	3	10	9	15	20	10	7	4	87
	9	3	4	5	6	13	19	7	18	10	13	98
	10	6	10	24	21	38	59	62	57	98	106	481
	
	Sum	33	70	80	90	110	148	141	121	147	173	1,113

**Table 14a t14a-jresv63an2p101_a1b:** 

cos *θ*												
	*k*	1	2	3	4	5	6	7	8	9	10	Sum
	*i*											
	
	1	……….	……….	……….	……….	……….	……….	……….	……….	……….	……….	0
	2	3	3	11	5	15	16	15	11	3	……….	82
	3	1	……….	1	1	3	2	2	8	13	23	54
	4	……….	……….	1	1	……….	2	4	3	2	3	16
*E*	5	……….	……….	……….	1	2	1	2	2	1	2	11
	6	……….	1	1	1	5	2	1	7	1	4	23
	7	……….	……….	……….	2	3	1	3	1	3	5	18
	8	……….	……….	2	2	2	3	1	……….	5	3	18
	9	……….	1	……….	2	2	4	6	3	5	7	30
	10	2	4	6	10	14	22	30	31	50	50	219
	
	Sum	6	9	22	25	46	53	64	66	83	97	471

**Table 14b t14b-jresv63an2p101_a1b:** 

cos *θ*												
	*k*	1	2	3	4	5	6	7	8	9	10	Sum
	*i*											
	
	1	……….	……….	……….	……….	……….	……….	……….	……….	……….	……….	0
	2	4	3	12	6	16	18	16	17	3	……….	95
	3	1	……….	1	1	3	4	2	10	16	28	66
	4	……….	……….	1	1	……….	2	4	3	2	3	16
*E*	5	……….	……….	……….	1	2	1	2	2	1	3	12
	6	……….	1	1	1	5	2	3	7	2	4	26
	7	……….	1	……….	2	3	1	3	3	3	5	21
	8	……….	……….	2	2	3	3	1	1	5	3	20
	9	……….	1	……….	3	3	4	6	3	5	8	33
	10	2	4	7	10	14	22	31	33	51	51	225
	
	Sum	7	10	24	27	49	57	68	79	88	105	514

**Table 15a t15a-jresv63an2p101_a1b:** 

cos *θ*												
	*k*	1	2	3	4	5	6	7	8	9	10	Sum
	*i*											
	
	1	……….	……….	……….	……….	……….	……….	……….	……….	……….	……….	0
	2	……….	2	2	3	4	2	5	3	2	……….	23
	3	……….	……….	2	3	……….	……….	……….	1	6	21	33
	4	……….	1	1	……….	1	……….	2	2	2	4	13
*E*	5	……….	……….	……….	……….	2	……….	……….	3	1	2	8
	6	……….	1	3	……….	2	3	……….	2	4	1	16
	7	……….	……….	1	……….	1	3	3	1	3	1	13
	8	……….	1	……….	4	1	7	3	3	3	……….	22
	9	……….	……….	……….	1	2	3	5	3	9	10	33
	10	2	5	6	14	17	26	17	23	28	21	159
	
	Sum	2	10	15	25	30	44	35	41	58	60	320

**Table 15b t15b-jresv63an2p101_a1b:** 

cos *θ*												
	*k*	1	2	3	4	5	6	7	8	9	10	Sum
	*i*											
	
	1	……….	……….	……….	……….	……….	……….	……….	……….	……….	……….	0
	2	1	2	3	5	8	2	5	6	3	1	36
	3	……….	1	2	4	1	1	……….	2	11	27	49
	4	……….	1	1	……….	1	3	4	4	2	4	20
*E*	5	……….	……….	……….	2	4	1	3	3	2	4	19
	6	……….	1	3	1	4	3	……….	3	4	1	20
	7	……….	……….	2	……….	2	4	4	1	3	2	18
	8	……….	1	1	4	1	7	3	4	3	2	26
	9	……….	……….	……….	3	3	3	6	3	10	12	40
	10	2	6	7	19	18	29	20	24	30	27	182
	
	Sum	3	12	19	38	42	53	45	50	68	80	410

**Table 15c t15c-jresv63an2p101_a1b:** 

cos *θ*												
	*k*	1	2	3	4	5	6	7	8	9	10	Sum
	*i*											
	
	1	……….	……….	……….	……….	……….	……….	……….	……….	……….	……….	0
	2	3	4	4	6	8	2	5	7	4	1	44
	3	……….	1	2	5	1	2	1	4	14	33	63
	4	……….	1	1	……….	1	3	4	5	2	3	20
*E*	5	……….	1	……….	3	4	1	3	3	3	3	21
	6	……….	1	3	2	4	3	……….	3	4	……….	20
	7	……….	……….	2	……….	2	4	4	2	3	2	19
	8	……….	……….	1	……….	1	3	1	4	3	2	15
	9	……….	……….	……….	2	3	2	4	2	5	7	25
	10	2	7	6	14	15	30	18	21	28	24	165
	
	Sum	5	15	19	32	39	50	40	51	66	75	392

**Table 16a t16a-jresv63an2p101_a1b:** 

cos *θ*												
	*k*	1	2	3	4	5	6	7	8	9	10	Sum
	*i*											
	
	1	……….	……….	……….	……….	……….	……….	……….	……….	……….	……….	0
	2	……….	3	4	4	6	3	4	2	1	……….	27
	3	……….	……….	……….	3	……….	1	……….	1	7	21	33
	4	……….	……….	……….	……….	……….	……….	3	1	……….	2	6
*E*	5	……….	……….	……….	……….	1	1	1	1	1	1	6
	6	2	1	3	8	10	10	9	16	14	7	80
	7	……….	……….	1	1	4	6	5	8	8	11	44
	8	……….	1	1	2	2	1	5	2	4	5	23
	9	1	……….	1	3	2	5	5	5	4	6	32
	10	1	3	2	11	14	15	15	13	25	22	121
	
	Sum	4	8	12	32	39	42	47	49	64	75	372

**Table 16b t16b-jresv63an2p101_a1b:** 

cos *θ*												
	*k*	1	2	3	4	5	6	7	8	9	10	Sum
	*i*											
	
	1	……….	……….	……….	……….	……….	……….	……….	……….	……….	……….	0
	2	1	6	10	7	16	10	13	13	2	……….	78
	3	……….	……….	……….	……….	3	1	2	7	13	13	39
	4	1	……….	……….	1	……….	2	……….	1	2	1	8
*E*	5	……….	1	……….	……….	……….	……….	4	1	2	……….	8
	6	2	1	3	8	9	7	8	17	13	10	78
	7	……….	……….	2	4	2	9	6	10	5	14	52
	8	……….	……….	1	6	3	2	6	7	4	6	35
	9	1	……….	……….	5	6	6	6	1	4	9	38
	10	2	4	3	12	11	23	20	23	27	28	153
	
	Sum	7	12	19	43	50	60	65	80	72	81	489

**Table 16c t16c-jresv63an2p101_a1b:** 

cos *θ*												
	*k*	1	2	3	4	5	6	7	8	9	10	Sum
	*i*											
	
	1	……….	……….	……….	……….	……….	……….	……….	……….	……….	……….	0
	2	5	8	11	12	14	6	13	13	4	2	88
	3	……….	1	3	9	4	3	5	14	34	56	129
	4	2	1	……….	……….	3	1	8	5	2	4	26
*E*	5	……….	1	……….	3	2	3	4	3	4	4	24
	6	……….	……….	……….	2	2	4	3	4	10	1	28
	7	……….	……….	……….	2	3	4	4	2	4	4	23
	8	……….	……….	2	1	3	4	3	1	5	4	23
	9	1	……….	2	2	3	5	2	4	8	9	36
	10	2	1	2	13	10	15	22	15	21	16	117
	
	Sum	10	14	20	44	44	45	64	61	92	100	494

**Table 16d t16d-jresv63an2p101_a1b:** 

cos *θ*												
	*k*	1	2	3	4	5	6	7	8	9	10	Sum
	*i*											
	
	1	……….	……….	……….	……….	……….	……….	……….	……….	……….	……….	0
	2	……….	3	4	4	6	3	4	2	1	……….	27
	3	……….	……….	……….	3	……….	1	……….	1	7	21	33
	4	……….	……….	……….	……….	……….	……….	3	1	……….	2	6
*E*	5	……….	……….	……….	……….	1	1	1	1	1	1	6
	6	……….	1	……….	1	1	3	1	2	3	1	13
	7	……….	……….	……….	……….	1	2	……….	1	2	2	8
	8	……….	……….	……….	1	2	1	1	……….	……….	1	6
	9	1	……….	……….	……….	1	3	1	1	4	3	14
	10	1	1	1	8	9	11	14	8	15	11	79
	
	Sum	2	5	5	17	21	25	25	17	33	42	192

## Footnotes

2 Figures in brackets indicate the literature references at the end of this paper.

3 See, however, sec. 7.8.

4 For a survey of the neutron cross section situation in relation to transport calculations, see the recent book by Goldstein [7].

5 Due to theoretical and experimental uncertainties, the cross sections given in this publication are subject to revision. We understand that Dr. Goldstein and his associates are now engaged in making an improved evaluation.

6 This assumption is open to doubt at energies above, say, 5 Mev. See Goldstein, Zweifel and Foster [10].

7 For a derivation, see, e.g., Bethe [12].

8 See also figure 5.

9 See, e.g., reference [13] for background and bibliography.

10 See, e.g., the article by J. Todd in reference [13c].

11 Using formula (5.9), noting that the range of the angular distribution (in cosθ*_c_*) is *a* = 2, and using the differential cross sections as shown in figure 3, we find that the average number of random number pairs that must be tested to make a selection has the following values: 6.8 at 14 Mev, 4.3 at 9 Mev, 2.2 at 6 Mev, 2.6 at 3 Mev, 2.8 at 1 Mev, and 1.03 at 0.3 Mev. The sampling efficiency at higher energies could be improved by more elaborate schemes, but we have not considered this worthwhile.

12 Crude estimates of the differential albedo can be obtained by counting the neutrons that are reflected with energies and obliquities in specified energy-angle intervals. The statistical accuracy of such estimates is given by formulas analogous to those for the number albedo.

13 Actually one does not know the exact value of *A_N_*, but an estimate of var $\left(A_{N}^{*}\right)$ is obtained through replacing *A_N_* in (5.27) by $A_{N}^{*}$, and *N*_0_ by *N*_0_−1 in order to make the estimate unbiased.

14 In fact, the distribution of $A_{N}^{*}$ is the N_0_-fold convolution of a binomial distribution with itself, and is very closely approximated by a Gaussian even for *N*_0_ as low as 10. The numerical results for the energy albedo indicate that the distribution of $A_{E}^{*}$ is a reasonable approximation to a Gaussian for *N*_0_≈100.

15 This case also arises in the comparison of the reflection of neutrons after 1, 2, … *n* collisions.

16 We have set *k*_1_=11, *k*_2_=15, and *k*_3_=13.

17 The program was based in part on an earlier code [15] developed at the Westinghouse Atomic Power Division. The authors are indebted to Dr. B. H. Mount and Mrs. Heidi Kuehn who very graciously made their code available to us.

18 To be reported in a later publication.

19 In sec. 5.3. the albedo estimates were indicated by *A*N* and *A*E* in order to distinguish them from the true values *A_N_* and *A_E_* In the following, we shall ignore this distinction and simply use *A_N_* and *A_E_* to label the Monte Carlo results.

20 The most accurate estimation procedure was found to be one based on the “cumulative albedo” $\int_{0}^{E}dE\prime \int_{\pi /2}^{\pi }\sin\;\theta d\theta A(E\prime ,\theta ;E_{0},\theta_{0})$, which can be obtained from the differential-albedo tables for *E_i_=i/*10*E*_0_,* i*=l, 2. … 10, and by interpolation for other values of *E.* It was also necessary to take into account the characteristics of the spectrum of the single-scattering albedo, in order to obtain good accuracy.

21 Some contributions from orders beyond the twentieth would have occurred if our calculations had not been cut off at an energy of 0.5 ev.

22 The histrograms are a somewhat crude estimate of the angular distribution, because each angular element was obtained by multiplying the differential element of the corresponding table in the app. C by the midpoint of the appropriate energy interval and then summing over-all energy intervals.

23 For the analysis of an albedo experiment with a broad source spectrum (such as the Po-Be source used by Strickler, Gilbert, and Auxier [16]), it might be preferable to make a Monte Carlo calculation with a realistic source spectrum instead of combining the results of calculations for monoenergetic sources.

## 8. Appendix A. Integrals Over the Differential Single-Scattering Albedo

### 8.1. Energy Spectrum

The evaluation of (4.15) leads to the following expression for the energy spectrum of neutrons reflected from a semi-infinite medium after exactly one collision:

$$A^{\left(1\right)}(E;E_{0},\theta_{0})=\sum_{j=1}^{3}p_{j}(E_{0})F_{j}(E;E_{0})\;\{U(a_{j},b_{j})-kV(a_{j},b_{j})\},$$ (A.1)

where

$$\begin{array}{l} a_{j}=\cos\;(\alpha_{j}+\theta_{0})\\ b_{j}=\cos\;(\alpha_{j}-\theta_{0})\\ k=(\lambda_{0}/\lambda )\;\cos\;\theta_{0}, \end{array}$$ (A.2)

and where *U*(*a_j_,b_j_*) and *V*(*a_j_,b_j_*) are defined as follows:

1. If $E_{0}{\left[\frac{\sqrt{M_{j}^{2}K_{j}^{2}-\cos^{2}\;\theta_{0}}+\sin\;\theta_{0}}{M_{j}+1}\right]}^{2}\leq E\leq E_{0},$
  $$U(a_{j},b_{j})=V(a_{j},b_{j})=0.$$ (A.3)
2. If $E_{0}{\left[\frac{\sqrt{M_{j}^{2}K_{j}^{2}-\cos^{2}\;\theta_{0}}-\sin\;\theta_{0}}{M_{j}+1}\right]}^{2}\leq E\leq E_{0}{\left[\frac{\sqrt{M_{j}^{2}K_{j}^{2}-\cos^{2}\;\theta_{0}}+\sin\;\theta_{0}}{M_{j}+1}\right]}^{2},$
  $$U(a_{j},b_{j})=\frac{1}{2}-\frac{1}{\pi }\sin^{-1}\frac{b_{j}+a_{j}}{b_{j}-a_{j}};$$ (A.4)
  $$\begin{array}{l} \text{if}\;k<b_{j},\;V(a_{j},b_{j})=\frac{1}{\pi \sqrt{\left(b_{j}-k)(k-a_{j}\right)}}\{\log(b_{j}-a_{j})k-\log[(b_{j}+a_{j})k-2a_{j}b_{j}-2\sqrt{a_{j}b_{j}(b_{j}-k)(a_{j}-k)}]\};\\ \text{if}\;k=b_{j},\;V(a_{j},b_{j})=\frac{2}{\pi (b_{j}-a_{j})}\sqrt{\frac{-a_{j}}{b_{j}}};\\ \text{if}\;k>b_{j},\;V(a_{j},b_{j})=\frac{1}{\sqrt{\left(k-b_{j})(k-a_{j}\right)}}\left\{\frac{1}{2}-\frac{1}{\pi }\sin^{-1}\left[\frac{(b_{j}+a_{j})k-2a_{j}b_{j}}{(b_{j}-a_{j})k}\right]\right\}. \end{array}\}$$ (A.5)
3. If $E_{0}{\left[\frac{M_{j}K_{j}-1}{M_{j}+1}\right]}^{2}\leq E\leq E_{0}[\frac{\sqrt{M_{j}^{2}K_{j}^{2}-\cos^{2}\;\theta_{0}}-\sin\;\theta_{0}}{M_{j}+1}{]}^{2}$
  $$U(a_{j},b_{j})=1$$ (A.6)
  $$V(a_{j},b_{j})=\frac{1}{\sqrt{\left(k-b_{j})(k-a_{j}\right)}}$$ (A.7)
4. If $E\leq E_{0}{\left[\frac{M_{j}K_{j}-1}{M_{j}+1}\right]}^{2}$
  $$U(a_{j},b_{j})=V(a_{j},b_{j})=0.$$ (A.8)

In certain limiting cases, the formula for the energy spectrum becomes much simpler than the above expressions. For perpendicular incidence, the energy must lie in the range

$$E_{0}{\left[\frac{M_{j}K_{j}-1}{M_{j}+1}\right]}^{2}\leq E\leq E_{0}\frac{M_{j}^{2}K_{j}^{2}-1}{{\left(M_{j}+1\right)}^{2}},$$ (A.9)

and the spectrum is

$$A^{\left(1\right)}(E;E_{0},0)=\sum_{j=1}^{3}p_{j}(E_{0})F_{j}(E;E_{0})\frac{1}{1-\frac{\lambda_{0}}{\lambda \;\cos\;\alpha_{j}}}.$$ (A.10)

For grazing incidence (*θ*_0_→*π*/2), the energy range is

$$E_{0}{\left[\frac{M_{j}K_{j}-1}{M_{j}+1}\right]}^{2}\leq E\leq E_{0}{\left[\frac{M_{j}K_{j}+1}{M_{j}+1}\right]}^{2}$$ (A.11)

and the spectrum is

$$A^{\left(1\right)}\left(E;E_{0},\frac{\pi }{2}\right)=\sum_{j=1}^{3}p_{j}(E_{0})\frac{1}{2}F_{j}(E;E_{0}).$$ (A.12)

Figure 15 shows energy spectra of neutrons reflected after a single scattering from a semiinfinite medium of pure oxygen *(E*_0_=1 Mev, *θ*_0_=0°, 45°, and 90°). These spectra were obtained by numerical evaluation of the factor

$$F_{2}(E;E_{0})\left\{U(\alpha_{2},b_{2})-kV(\alpha_{2},b_{2})\right\}$$

in (A.1). They are characterized by a peak at the low-energy limit which is rather independent of the source obliquity *θ*_0_. In addition there is another peak at the high-energy limit in the case of grazing incidence.

For perpendicular and grazing incidence the spectrum for reflection from water can be derived very simply from the corresponding spectrum for oxygen. For *θ*_0_=0°, reflection from hydrogen is impossible after one scattering, and it is sufficient to renormalize the oxgyen spectrum in figure 15, multiplying it by *p*_2_(*E*_0_)=0.485. For *θ*_0_=90°, one must not only normalize the oxygen contribution but also add a hydrogen contribution which is energy-independent and has the value *p_1_*(*E*_0_)½*F*_1_(*E*;*E*_0_) = 0.515 (1/2*E*_0_)=0.2575.

### 8.2. Angular Distribution

The angular distribution of neutrons reflected after one collision is even more complicated than their energy spectrum. We shall confine ourselves to the discussion of some limiting cases. The characteristics of the angular distribution are expressible in terms of the integrals

$$h_{j}\left(\theta ;E_{0},\theta_{0}\right)=\int \frac{H_{j}\left(E,\theta ;E_{0},\theta_{0}\right)\;\cos\;\theta }{\cos\;\theta -\left(\lambda_{0}/\lambda \right)\;\cos\;\theta_{0}}dE$$ (A.13)

which are needed for the evaluation of (4.16). The angular distribution of reflected energy, $A_{E}^{\left(1\right)}\left(\theta ;E_{0},\theta_{0}\right)$ depends on the integrals

$$h_{Ej}\left(\theta ;E_{0},\theta_{0}\right)=\int \frac{H_{j}\left(E,\theta ,E_{0};\theta_{0}\right)\;\cos\;\theta }{\cos\;\theta -\left(\lambda_{0}/\lambda \right)\;\cos\;\theta_{0}}EdE.$$ (A.14)

The integrations in (A.13) and (A.14) are facilitated by changing the variable of integration from *E* to cos *α_j_*, noting that

$$E=E_{0}{\left[\frac{\cos\alpha_{j}+\sqrt{\cos^{2}\;\alpha_{j}+M_{j}^{2}K_{j}^{2}-1}}{M_{j}+1}\right]}^{2}$$ (A.15)

and that

$$\frac{dE}{d\;\cos\;\alpha_{j}}=\frac{2E_{0}}{{\left(M_{j}+1\right)}^{2}}\left\{2\;\cos\alpha_{j}+\sqrt{M_{j}^{2}K_{j}^{2}-\sin^{2}\alpha_{j}}+\frac{\cos^{2}\;\alpha_{j}}{\sqrt{M_{j}^{2}K_{j}^{2}-\sin^{2}\alpha_{j}}}\right\}.$$ (A.16)

We now take up limiting cases, and drop the index *j* for simplicity.

Case (1):

Heavy nucleus, *M* ≫ 1
Perpendicular incidence, *θ*_0_ = 0°
Elastic scattering, *K* = l.

Note that

$$\begin{array}{l} \begin{array}{ll} E\approx E_{0}, & \lambda \approx \lambda_{0}, \end{array}\\ \frac{dE}{d\;\cos\;\alpha }\approx \frac{2E_{0}}{M},\\ \begin{array}{ll} \theta_{c}\approx \alpha , & F\left(E;E_{0}\right)\approx \frac{M}{2E_{0}}f\left(\alpha ;E_{0}\right) \end{array} \end{array}\}$$ (A.17)

Substituting expression (4.13) for *H*(*E*,*θ*; *E*_0_,0), we find that

$$h\left(\theta ;E_{0},0\right)\approx \frac{\cos\;\theta f\left(\alpha ;E_{0}\right)}{\cos\;\theta -1},$$ (A.18)

and that

$$h_{E}\left(\theta ;E_{0},0\right)\approx E_{0}h\left(\theta ;E_{0},0\right)$$ (A.19)

Case (2):

Heavy nucleus, M≫1
Grazing incidence, *θ*_0_= 90°
Elastic scattering, *K* = 1.

The approximations (A.17) may again be used. For $H\left(E,\theta ;E_{0},\frac{\pi }{2}\right)$ we substitute expression (4.14), the result being

$$h\left(\theta ;E_{0},\frac{\pi }{2}\right)=\frac{1}{\pi }\int_{-\sin\;\theta }^{\sin\;\theta }\frac{f\left(\alpha ;E_{0}\right)d\;\cos\;\alpha }{\sqrt{\sin^{2}\theta -\cos^{2}\;\alpha }}.$$ (A.20)

Assuming for *f*(*α*;*E*_0_) a Legendre expansion of the type (3.9), with expansion coefficients *fl* (*E*_0_), we can integrate (A.20) and find that

$$h\left(\theta ;E_{0},\frac{\pi }{2}\right)=\sum_{l=0}^{\infty }\frac{4l+1}{2}f_{2l}\left(E_{0}\right){\left(-1\right)}^{e}\frac{\left(2l\right)!}{2^{2e}l!l!}P_{2l}\left(\cos\;\theta \right),$$ (A.21)

and that

$$h_{E}\left(\theta ;E_{0},\frac{\pi }{2}\right)\approx E_{0}h\left(\theta ;E_{0},\frac{\pi }{2}\right).$$ (A.22)

Case (3):

Hydrogen nucleus, *M* = 1
Grazing incidence, *θ*_0_ = 90°.

We have *f*(θ*_c_*;*E*_0_) = 1/2 and 0 ≤ *α* ≤ *π*/2. Using (A.14), (A.15), and (A.16), we find that

$$h\left(\theta ;E_{0},\frac{\pi }{2}\right)=\frac{2}{\pi }\int_{0}^{\sin\;\alpha }\frac{\cos\;\alpha d\;\cos\;\alpha }{\sqrt{\sin^{2}\theta -\cos^{2}\;\alpha }}=\frac{2}{\pi }\sin\;\theta ,$$ (A.23)

and that

$$h_{E}\left(\theta ;E_{0},\frac{\pi }{2}\right)=\frac{2E_{0}}{\pi }\int_{0}^{\sin\;\theta }\frac{\cos^{3}\;\alpha d\;\cos\;\alpha }{\sqrt{\sin^{2}\theta -\cos^{2}\;\alpha }}=\frac{4E_{0}}{3\pi }\sin^{3}\theta .$$ (A.24)

## 9. Appendix B. Pseudorandom Numbers Generated by Congruential Multiplication

### 9.1. Proof of Their Uniform Distribution

We consider the pseudorandom number sequence {ξ*_n_*= 2^−35^*x_n_*} (*n* = 0, 1,…, 2^33^−1) derived from a sequence of integers {*x_n_*} which is generated according to (5.4) or by the equivalent rule

$$x_{n}=c5^{kn}\;\mathrm{mod}\;2^{35},$$ (B.1)

where *c* and *k* are odd integers. The sequence {*ξ_n_*} consists of 2^33^ different numbers which are distributed with uniform density over the interval (0,1). This can be proved with the use of standard number-theoretical results [17]. However, Dr. Morris Newman^24^ has given a simple proof proceeding from first principles which we reproduce here in a somewhat amplified form.

The uniformity of the distribution of the pseudorandom numbers obtained by congruential multiplication is a consequence of the following theorem.

Theorem: *The sequence of integers*

$$\left\{x_{0},x_{1},x_{2},\ldots ,x_{2}^{33}-1\right\}$$ (B.2)

*generated according to* (B.1), *is a permutation of the sequence*

$$\left\{1,5,9,\ldots ,2^{35}-3\right\}$$ (B.3)

*or of the sequence*

$$\left\{3,7,11,\ldots ,2^{35}-1\right\}$$ (B.4)

*depending on whether the starting number x*_0_ *= c has remainder 1 or 3 when divided by 4.*

The proof of this theorem is based on the following lemma:

*The integer* $5^{k2^{M}}-1$ *is divisible by* 2*^M^*^+2^ *but by no higher power of* 2, *if* k *is an odd integer*.

To see this we consider the factorization

$$5^{k2^{M}}-1=(5^{k2^{M-1}}+1)(5^{k^{2M-1}}-1)=(5^{k2^{M-1}}+1)(5^{k2^{M-2}}+1)\ldots (5^{k}+1)(5^{k}-1).$$ (B.5)

The factor

$$5^{k}-1={\left(1+4\right)}^{k}-1=4k+\sum_{j=2}^{k}\left(\begin{array}{c} k\\ j \end{array}\right)4^{j}$$ (B.6)

is divisible by 4 but by no higher power of 2. The other factors in (B.5) have the form

$$5^{k2^{m}}+1={\left(1+4\right)}^{k2^{m}}+1=2+\sum_{j=1}^{k2^{m}}\left(\begin{array}{c} k2^{m}\\ j \end{array}\right)4^{j},0\leq m\leq M-1$$ (B.7)

and are each divisible by 2 but by no higher power of 2. By combining all the factors we obtain the lemma.

We now consider the sequence {*c*5*^kn^*}, *n*=0,1, … 2^33^—1. The difference between any two terms in this sequence, say *c*5*^k^*^(^*^n+n^*^′)^ and *c*5*^kn^*, can be expressed as

$$\Delta (n,n\prime )=c5^{kn}(5^{kn\prime }-1).$$ (B.8)

We may set

$$n\prime =2^{a}b,$$ (B.9)

where *a* is an integer <2^33^, and where *b* is an odd integer. By application of the lemma it follows that 5*^kn^′ —* 1, and therefore Δ(*n,n*′), is divisible only by powers of 2 smaller than 2^35^. This implies that the integers *x_n_* in the sequence (B.2) are all different from each other.

It can be seen from (B.8) that the differences between the *x_n_*’s are all multiples of 4. Thus two different sequences {*x_n_*} can be formed, one of which is a permutation of (B.3) and the other of (B.4), depending on the starting number *c.* Each of the two sequences contains 2^33^ numbers, and there is no overlap between them, so that together they exhaust the 2^34^ odd integers <2^35^. This completes the proof of the theorem. Finally, we note that by computing the *x_n_*’s modulo 2*^N^* rather than modulo 2^35^, and by using a starting number *c*, that is not odd but contains a factor 2^γ^, one would obtain a sequence of *2^N^*^−2−γ^ different numbers.

### 9.2. Statistical Indications of Their Randomness

In order to be acceptable as a source of random numbers for a Monte Carlo calculation, the pseudorandom number sequence {*ξ_n_*} must fulfill the following requirements:

1. In any subsequence of reasonable length, such as would be used in generating neutron histories, the *ξ_n_*’s must be distributed with uniform density in the interval (0,1).
2. Successive *ξ_n_*’s must be uncorrelated.

These two conditions cannot be satisfied rigorously, but empirical statistical tests indicate that they are satisfied in very good approximation. For example, elaborate tests on 16,384 pseudorandom numbers have been reported in the memorandum mentioned in footnote 24.

We have made some rough-and-ready statistical tests on 237,000 pseudorandom numbers that were used to generate a set of 3,000 neutron histories.^25^ These numbers were divided into three sets, corresponding to the three types of pseudorandom number sequences (primary, secondary, and tertiary) described in section 5.4. Set *P* includes the numbers in the primary sequence, set *S* the numbers in all the secondary sequences, and set *T* the numbers in all the tertiary sequences. Separate tests were applied to each set. In order to gauge the uniformity of the distribution of the numbers, we computed the averages $\bar{\xi_{n}}$ and $\bar{\xi_{n}{\;}^{2}}$. In order to check on the lack of correlation between successive pseudorandom numbers we computed the mean square difference of successive numbers, $\bar{{\left(\xi_{n+1}-\xi_{n}\right)}^{2}}.$ As a further test of possible correlation, the algebraic sign of the differences between successive pseudorandom numbers was examined, and an index *t* was computed which was defined as the excess of positive over negative signs. Table B.1 compares the actual values of all these quantities with the values that one would expect if the pseudorandom numbers were truly random. We infer from the table that the pseudorandom numbers are sufficiently random for our purpose.

**Table B.1:** Analysis of the randomness of the pseudorandom numbers In order to indicate the significance of the deviations of the empirical from the expected values of the various statistics, the standard deviations of the empirical values are given. The pseudorandom numbers were obtained by starting a primary sequence with the initial value *c* = 16,743,785,845. The number of pseudorandom numbers in each set is indicated within brackets.

Statistic	Expected value	Empirical value		
		Set *P* [3,000]	Set *S* [220,776]	Set *T* [13,384]

$\bar{\xi_{n}}$	½	0.49402±0.00527	0.49955±0.00061	0.50233±0. 00250
$\bar{\xi_{n}^{2}}$	⅓	.32937±.00.544	.33359±.00064	.33580±.00258
$\bar{{\left(\xi_{n+1}-\xi_{n}\right)}^{2}}$	⅙	.16743±.00360	.16579±.00042	.16461±.00171
*t*	0	28 ±55	−638 ±470	34 ±116

## 10. Appendix C. Tables of Differential Albedo

The following tables are indexed by table 6.1 and described in section 6.2 of the text. [Table t1-jresv63an2p101_a1b] [Table t2a-jresv63an2p101_a1b] [Table t2b-jresv63an2p101_a1b] [Table t2c-jresv63an2p101_a1b] [Table t3a-jresv63an2p101_a1b] [Table t3b-jresv63an2p101_a1b] [Table t4a-jresv63an2p101_a1b] [Table t4b-jresv63an2p101_a1b] [Table t5a-jresv63an2p101_a1b] [Table t5b-jresv63an2p101_a1b] [Table t6a-jresv63an2p101_a1b] [Table t6b-jresv63an2p101_a1b] [Table t7a-jresv63an2p101_a1b] [Table t7b-jresv63an2p101_a1b] [Table t7c-jresv63an2p101_a1b] [Table t8a-jresv63an2p101_a1b] [Table t8b-jresv63an2p101_a1b] [Table t8c-jresv63an2p101_a1b] [Table t9a-jresv63an2p101_a1b] [Table t9b-jresv63an2p101_a1b] [Table t10a-jresv63an2p101_a1b] [Table t10b-jresv63an2p101_a1b] [Table t11a-jresv63an2p101_a1b] [Table t11b-jresv63an2p101_a1b] [Table t12a-jresv63an2p101_a1b] [Table t12b-jresv63an2p101_a1b] [Table t13-jresv63an2p101_a1b] [Table t14a-jresv63an2p101_a1b] [Table t14b-jresv63an2p101_a1b] [Table t15a-jresv63an2p101_a1b] [Table t15b-jresv63an2p101_a1b] [Table t15c-jresv63an2p101_a1b] [Table t16a-jresv63an2p101_a1b] [Table t16b-jresv63an2p101_a1b] [Table t16c-jresv63an2p101_a1b] [Table t16d-jresv63an2p101_a1b]
